# Identification and experimental validation of circular RNA-associated ceRNA networks in intrauterine adhesion

**DOI:** 10.3389/fgene.2025.1619698

**Published:** 2025-08-29

**Authors:** Yajie Chang, Rui Xiang, Qi Guo, Jing Li, Xiaolan Li, Zhi Zeng, Jintao Peng, Xiaoyan Liang

**Affiliations:** ^1^ Reproductive Medicine Center, The Sixth Affiliated Hospital, Sun Yat-sen University, Guangzhou, China; ^2^ GuangDong Engineering Technology Research Center of Fertility Preservation, Guangzhou, China; ^3^ Biomedical Innovation Center, The Sixth Affiliated Hospital, Sun Yat-sen University, Guangzhou, China

**Keywords:** circular RNA, hsa_circ_0000994, intrauterine adhesions, fibrosis, competing endogenous RNA

## Abstract

Intrauterine adhesion (IUA) is characterized by endometrial fibrosis, posing significant risks to women’s reproductive health and fertility. This study aimed to uncover a circRNA-associated ceRNA regulatory network relevant to intrauterine adhesion (IUA), thereby contributing to the understanding of its molecular pathogenesis. The expression data of circRNAs and mRNAs in endometrial tissues of IUA and normal controls were analyzed by RNA sequencing, and microRNAs (miRNAs) expression data was downloaded from GSE165321. Our analysis identified 44 differentially expressed (DE) circRNAs, 41 DEmiRNAs, and 640 DEmRNAs. A comprehensive circRNA-miRNA-mRNA network was constructed using Cytoscape. DEmRNAs were mainly enriched in extracellular matrix structural components, collagen fiber complexes. KEGG pathway analysis further implicated the NF-κB signaling pathway, apoptosis, and Notch signaling in IUA development. A protein-protein interaction network for ceRNA-associated mRNAs was developed through the STRING database, highlighting potential hub genes. To validate these transcriptomic findings, RT-qPCR confirmed significant upregulation of the two leading hub circRNAs, hsa_circ_0000439 and hsa_circ_0000994, in IUA samples compared to normal controls, with results showing consistency with RNA sequencing data (p < 0.05). Functional experiments demonstrated that silencing hsa_circ_0000994 with siRNA *in vitro* significantly decreased the expression levels of fibrosis markers α-SMA and COL1A1 in human endometrial stromal cells treated with TGF-β1. In conclusion, this study presents an in-depth transcriptomic analysis of the aberrantly expressed circRNAs, miRNAs, and mRNAs in endometrial tissues from patients with IUA, culminating in the establishment of a novel circRNA-miRNA-mRNA regulatory network. Hsa_circ_0000994 is likely to play a pivotal role in modulating fibrosis associated with IUA, and represents a promising candidate for targeted therapeutic approaches.

## 1 Introduction

Intrauterine adhesion (IUA) is a pathological condition characterized by endometrial fibrosis resulting from injury and impaired endometrial regeneration. Clinically, IUA manifests as reduced menstrual flow, secondary amenorrhea, infertility, and increased risks of miscarriage and abnormal placental implantation, profoundly impacting reproductive health of women. Despite the widespread use of transcervical resection of adhesions as the primary treatment, its efficacy is limited by high postoperative recurrence rates, particularly in severe cases, where recurrence rates can reach 62.5%, and pregnancy rates remain as low as 22.5%–33.3% (Lee et al., 2021). Effective therapeutic options for severe and recurrent IUA remain scarce, underscoring the urgent need to elucidate its pathogenic mechanisms and develop innovative prevention and treatment strategies (Ma et al., 2021).

Recent studies underscore the pivotal function of non-coding RNAs in various biological processes and pathological conditions. Non-coding RNAs, encompassing circular RNAs (circRNAs), long non-coding RNAs (lncRNAs), and microRNAs (miRNAs), are RNA species that, although not translated into proteins, play pivotal roles in cellular regulation and gene expression (Matsui and Corey, 2016). These molecules regulate various cellular functions, including mRNA translation, RNA processing, chromatin modification, and gene silencing (Beermann et al., 2016). CircRNAs distinguished by theirs covalently closed-loop configuration resulting from back-splicing, have attracted considerable interest owing to their remarkable stability and tissue-specific expression patterns (Della Bella et al., 2022; Kristensen et al., 2019; Chen et al., 2021). Functionally, circRNAs are integral components of competing endogenous RNA (ceRNA) networks, where they act as miRNA sponges to modulate gene expression (Liu et al., 2022). Although circRNAs have been implicated in the pathogenesis of fibrotic diseases, such as cardiac and liver fibrosis (Zhu et al., 2019; Zhou et al., 2018), their role in IUA remains largely unexplored.

Building on the ceRNA hypothesis, which describes interactions among lncRNAs, miRNAs, and mRNAs, the role of circRNAs within ceRNA networks and their specific contributions to the pathogenesis of IUA remain largely unexplored (Liu and Wang, 2022). To address this gap, the present study performs a comprehensive bioinformatics analysis of endometrial tissues from patients with IUA and normal controls (NC). Through the development of a circRNA–miRNA–mRNA interaction framework and identification of pivotal circRNAs, we seek to enhance insights into IUA pathophysiology and contribute to targeted therapeutic strategies.

## 2 Materials and methods

### 2.1 Sample collection and preparation

The research protocol underwent ethical review and received approval from the Ethics Committee of the Sixth Affiliated Hospital of Sun Yat-sen University (Approval No. 2022ZSLYEC-490). Prior to enrollment, written informed consent was secured from all participants, adhering to the ethical standards and institutional regulations. The work flow is shown in Supplementary Figure S1. Endometrial tissue samples were obtained during hysteroscopic procedures from two groups: patients diagnosed with intrauterine adhesion (IUA, n = 3) and a normal control (NC) group (n = 3), consisting of women without endometrial or uterine abnormalities, as confirmed by hysteroscopy and histopathology, who underwent hysteroscopic evaluation for non-intrauterine indications (e.g., tubal infertility assessment). All samples were collected during the proliferative phase of the menstrual cycle to limit hormonal fluctuations. Inclusion criteria for the IUA group were based on severe intrauterine adhesions, defined by an American Fertility Society score >9 (The American Fertility Society classifications, 1988). Exclusion criteria encompassed a history of tuberculosis (pulmonary or reproductive), metabolic or endocrine disorders, uterine malformations, or autoimmune diseases, as these conditions can independently alter endometrial physiology and gene expression, thereby introducing potential confounding factors unrelated to IUA. Participants in the NC group were selected based on regular menstrual cycles, normal uterine cavity structures, histopathologically confirmed absence of endometritis, and no history of endocrine or metabolic abnormalities or autoimmune conditions. None of the participants in either group received hormonal therapy within 3 months before sample collection.

Collected endometrial tissues were immediately rinsed with sterile physiological saline to remove blood and contaminants, then immersed in RNA later to preserve RNA integrity. Samples were subsequently stored in liquid nitrogen at −80°C until further processing.

### 2.2 RNA extraction, library preparation, and circRNA and mRNA sequencing

Total RNA was isolated from endometrial tissues of both IUA (n = 3) and NC groups (n = 3) using established laboratory procedures. Ribosomal RNA was selectively removed through the Epicentre Ribo-zero™ rRNA Removal Kit (Epicentre, United States), in accordance with the manufacturer’s instructions. The integrity and quality of RNA samples were assessed using the Agilent Technologies Bioanalyzer 2100 system (United States), with all samples meeting the requirement of a RIN value of 7 or higher. To further demonstrate the reliability and internal consistency of our RNA sequencing data despite the small sample size, we performed correlation analyses.

For sequencing, libraries specific to circRNA and mRNA were prepared following the protocols of the NEBNext^®^ Ultra™ Directional RNA Library Prep Kit and NEBNext^®^ Multiplex Small RNA Library Prep Set for Illumina^®^ (NEB, United States). Residual rRNA fragments were eliminated using ethanol precipitation. The libraries were prepared for sequencing using the TruSeq SR Cluster Kit v3-cBot-HS (Illumina, United States) and run on the Illumina Hiseq 4000 system, producing paired-end reads of 150 bp in length.

### 2.3 Data retrieval and differential expression profiling

Gene-specific read counts were calculated using HTSeq v0.13.5. To standardize the expression levels of circRNAs and mRNAs derived from sequencing data, the fragments per kilobase of transcript per million mapped reads (FPKM) normalization method was employed. The miRNA expression profiles for IUA patients (n = 3) and healthy controls (n = 3) were retrieved from the GEO dataset (GSE165321). Differential expression of circRNAs (DEcircRNAs), miRNAs (DEmiRNAs), and mRNAs (DEmRNAs) was determined using the DESeq2 package in R. To ensure robust identification of differentially expressed genes, a |log2 fold change| exceeding 1, coupled with an adjusted *p* < 0.05, was used as the threshold for significance.

### 2.4 Identification of enriched functions and pathways

To further analyze DEmRNAs, we used the R package “clusterProfiler” to conduct functional enrichment analyses for GO terms and KEGG pathways. The enrichment outcomes were visualized through the “goplot” and “enrichplot” R packages. Statistical significance was assessed using Fisher’s exact test, with a threshold of adjust *p* < 0.05.

### 2.5 Constructing the ceRNA network

The circBase (http://www.circbase.org/) and the Cancer-Specific CircRNA Database (CSCD, http://gb.whu.edu.cn/CSCD/) were utilized to screen for DEcircRNAs-target miRNAs (MRE). For enhanced precision, the acquired miRNAs (MRE) were intersected with the DEmiRNAs obtained from the GSE165321 dataset. Based on the miRNA data downloaded from two target gene prediction websites, targetScan (http://www.targetscan.org) and miRDB (http://www.mirdb.org/), we used Perl language to predict their target mRNAs. The miRNA target mRNAs that can be found in both databases are selected. Then we intersected them with DEmRNAs from circRNA Sequencing to obtain the final DemRNAs. We employed Cytoscape (v3.7) to build and visually represent the ceRNA network involving circRNA, miRNA, and mRNA, based on the ceRNA hypothesis.

### 2.6 Hub gene identification and protein-protein interaction network analysis

To investigate potential protein-level interactions among the DEmRNAs, we performed a protein-protein interaction (PPI) analysis using the STRING database (https://string-db.org/cgi/input.pl). Cytoscape (version 3.7) was employed to depict the protein-protein interaction network graphically. Central node proteins (hub proteins) with high connectivity in the network were selected based on their connectivity degrees. Consequently, we identified the top 10 hub genes.

### 2.7 Real-time quantitative polymerase chain reaction (RT-qPCR) validation

The expression levels of the two most significant circRNAs, hsa_circ_0000439 and hsa_circ_0000994, were confirmed in IUA (n = 11) and NC samples (n = 11) using RT-qPCR. Total RNA was extracted using the RNeasy Mini Kit (Qiagen, Germany), with subsequent cDNA synthesis conducted using the SuperScript III First-Strand Synthesis System (Life Technologies, United States). RT-qPCR was carried out on the AB StepOnePlus platform (Applied Biosystems, United States) with TB Green Premix EX Taq II (Takara, China), and β-actin was used as reference gene. Primer sequences were generated with Oligo7 software (Table 1), and all reactions were performed in triplicate to ensure experimental reproducibility.

**TABLE 1:** Primer sequence used for the qRT-PCR.

Gene	Sequence (5′–3′)
*hsa_circ_0000994*	FOR: AAA​CCA​TCG​AAG​GGA​CTG​CC
	REV: AAC​TGT​CAC​AAC​CTA​ACA​ATT​TCA​T
*hsa_circ_0000439*	FOR: TCT​ATG​CAA​ATA​TGA​GGA​TGG​TTC​A
	REV: CGT​AGA​CTG​AGG​CAG​TCC​TTT
*β-actin*	FOR: CAT​GTA​CGT​TGC​TAT​CCA​GGC
	REV: CTC​CTT​AAT​GTC​ACG​CAC​GAT
*COL1A1*	FOR: TGC​TCG​TGG​AAA​TGA​TGG​TG
	REV: GGA GCA CCA TTG GCA CCT TT
*αSMA*	FOR: AAT​ACT​CGG​TGT​GGA​TCG​GC
	REV: GTT​TAC​GAT​GGC​AGC​AAC​GG
*siMOCK*	FOR: CAG​CTA​CTG​TCG​ACT​TAC​ATT
	REV: AAT​GTA​AGT​CGA​CAG​TAG​CTG
siCircRNA 1	FOR: GCA​UCU​CAG​CAA​UGU​CAA​A
	REV: UUU​GAC​AUU​GCU​GAG​AUG​C
siCircRNA 2	FOR:GGAGCUCGAGGAAAUGUUA
	REV: UAA​CAU​UUC​CUC​GAG​CUC​C
siCircRNA 3	FOR:GGAUUUCAUCUGUUAGUUA
	REV: UAA​CUA​ACA​GAU​GAA​AUC​C

### 2.8 Cell culture and construction of IUA fibrosis cell model using HESCs

Human endometrial stromal cells (HESCs; OLC-0712, Guangzhou Shuangquan Biotech, China) were cultured in complete medium under standard conditions and passaged at 80% confluence. HESCs were plated into 96-well plates at a concentration of 5 × 10^3^ cells per well and exposed to varying doses of TGF-β1 (5, 10, 20 ng/mL) for 72 h to establish an *in vitro* cell model of IUA. Based on the effects observed at different TGF-β1 concentrations, the optimal concentration was selected for further experiments.

### 2.9 Transfection, and siRNA knockdown and real-time polymerase chain reaction (RT-qPCR) validation

For transfection, model cells were plated in 6-well plates at a density of 1 × 10^7^ cells per well for transfection. Transfection complexes were prepared by combining 20 μg hsa-circ-0000994 siRNA (si1, si2, and si3) with Lipofectamine 2000 (Lip2000, GZYXbio, China) in serum-free medium, following the manufacturer’s protocol. Knockdown efficiency was evaluated by extracting total RNA from transfected cells, reverse-transcribing it to cDNA, and performing RT-qPCR with β-actin as reference gene. To quantify gene expression, we employed the 2^−ΔΔCT^ method, which enables comparison of relative transcript levels across samples. Fibrotic markers, α-SMA and COL1A1, were evaluated by RT-qPCR. Each experiment was conducted with three independent replicates to ensure reproducibility.

### 2.10 Statistical analysis

All statistical analyses were conducted with R software (4.2.0) and GraphPad Prism (9.5.0). The Shapiro-Wilk test was applied to assess the normality of continuous variables. For data meeting the normal distribution assumption, the differences between groups were evaluated using the Student’s t-test. For data that did not follow a normal distribution, the Mann-Whitney U test was applied, while Categorical variables were analyzed using the chi-square test. RT-qPCR results were analyzed via paired t-tests to assess group-level differences. In the case of comparisons among multiple groups, a one-way ANOVA was employed to assess group differences. Statistical significance was established based on an adjusted p-value threshold of less than 0.05, indicating reliable results.

## 3 Results

### 3.1 Differential expression profiling of circRNAs, miRNAs, and mRNAs in IUA

**FIGURE 1:**
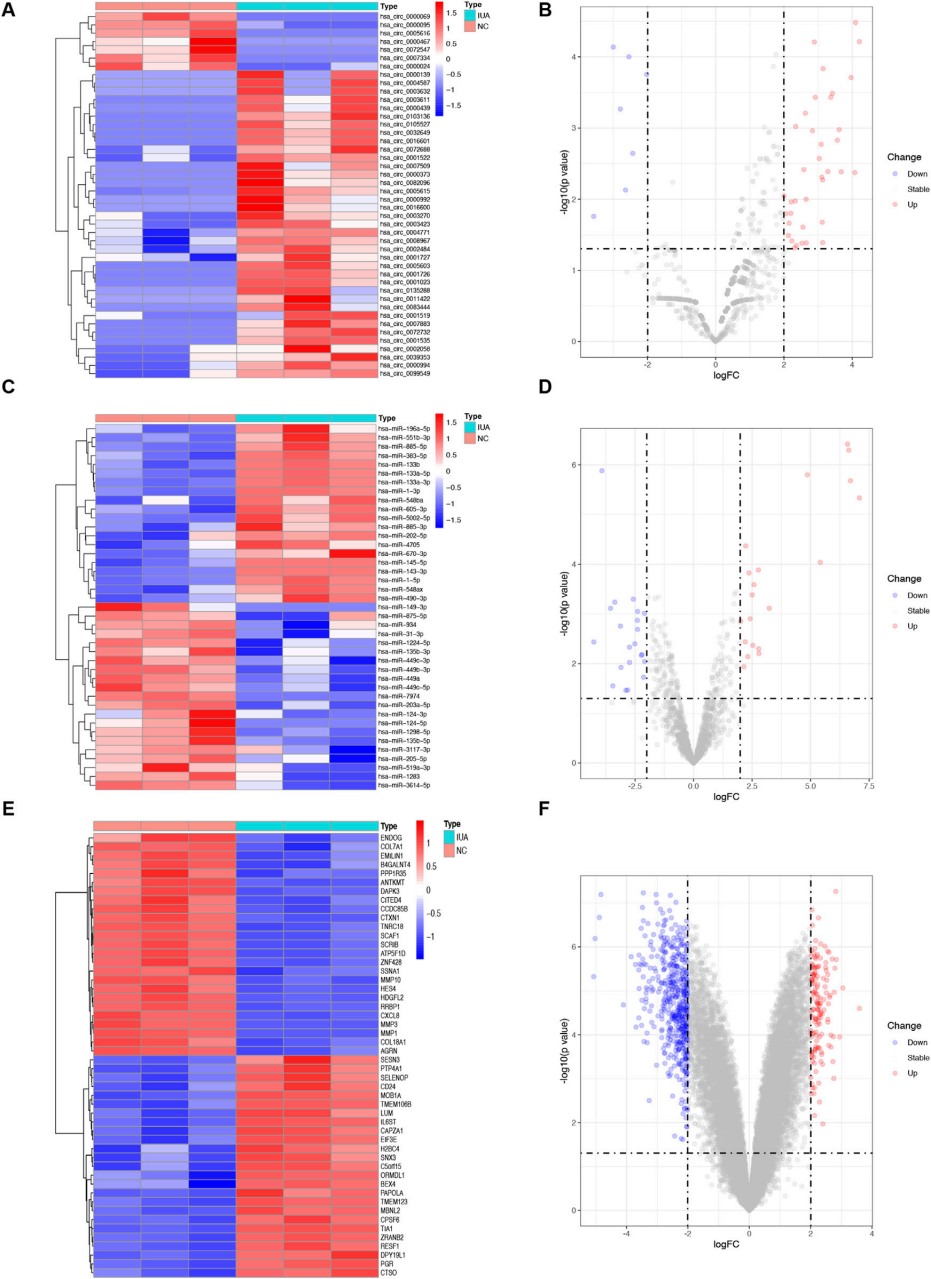
Differential expression of circRNAs (DEcircRNAs), miRNAs (DEmiRNAs), and mRNAs (DEmRNAs) between intrauterine adhesion (n = 3) and normal control (n = 3). **(A,B)** Heatmap and volcano plot of 44 DEcircRNAs. **(C,D)** Heatmap and volcano plot of 41 DEmiRNAs. **(E, F)** Heatmap and volcano plot of the top 50 DEmRNAs. The filtering criteria used were *p*-value <0.05 and | log_2_ fold change (FC) | > 1. IUA, intrauterine adhesion; NC, normal control; DEcircRNAs, differentially expressed circRNAs; DEmiRNAs, differentially expressed miRNAs; DEmRNAs, differentially expressed mRNAs.

DEcircRNAs, DEmRNAs, and DEmiRNAs were identified using the RNA sequencing and the GSE165321 dataset, comparing IUA and NC groupss. In total, 3,034 circRNAs, 1,631 miRNAs, and 16,324 mRNAs were detected. We identified 44 DEcircRNAs (37 upregulated, 7 downregulated; Figures 1A, B; Supplementary Table S1), 41 DEmiRNAs (20 upregulated, 21 downregulated; Figures 1C, D; Supplementary Table S2), and 640 DEmRNAs (147 upregulated, 493 downregulated; Figures 1E, F; Supplementary Table S3). The correlation heatmap (Supplementary Figure S2) demonstrated good reproducibility and clear biological distinction between groups, supporting the validity and reliability of our transcriptomic profiling despite the limited sample size.

### 3.2 GO and KEGG enrichment analysis of DEmRNAs

**FIGURE 2:**
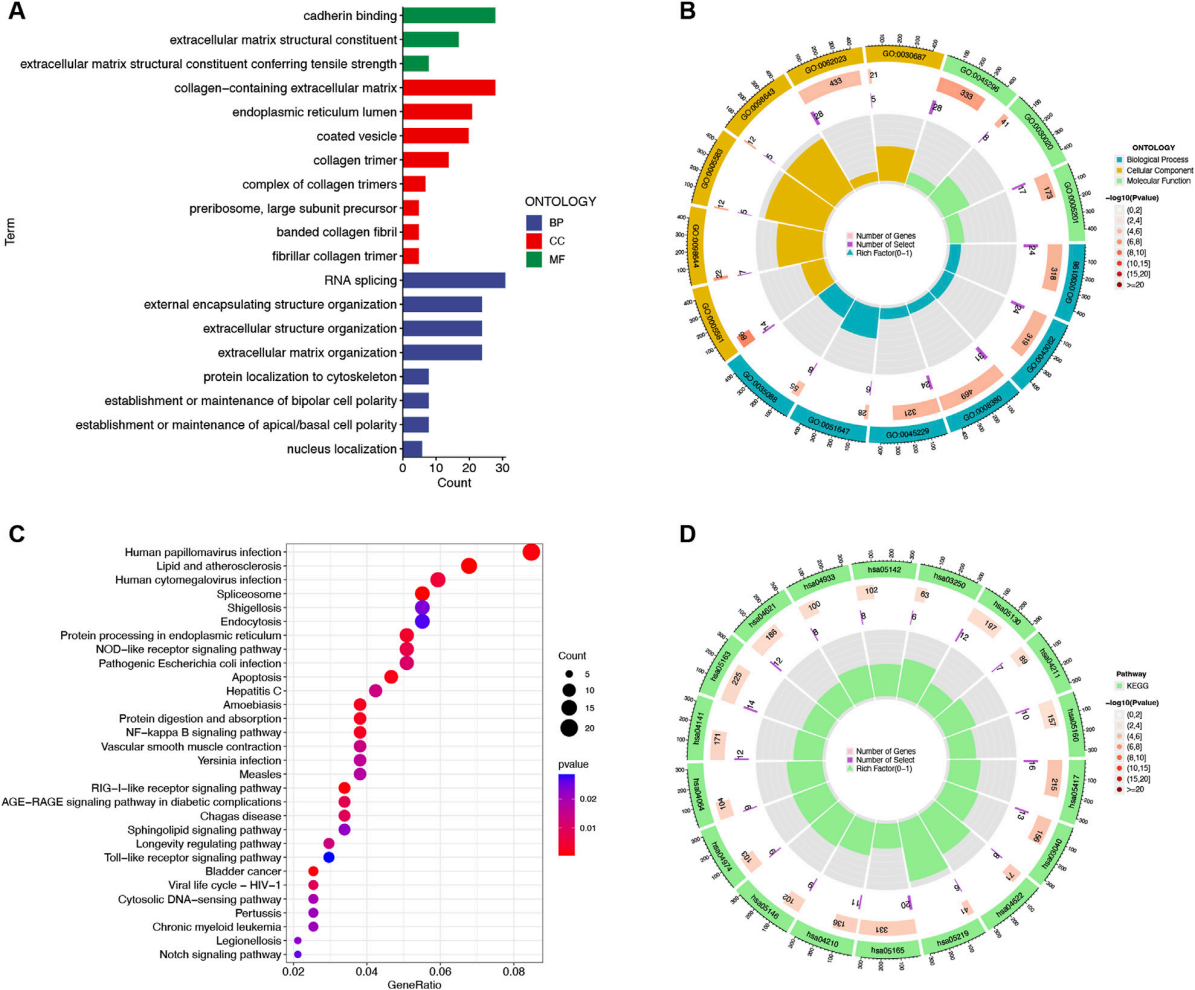
Functional enrichment analysis of 640 differentially expressed mRNAs between intrauterine adhesion and normal control. **(A,B)** Histogram and circle plots of Gene Ontology (GO) term enrichment analysis. **(C,D)** Bubble and circle plots of the Kyoto Encyclopedia of Genes and Genomes (KEGG) pathway analysis. BP, Biological Process; CC, Cellular Component; MF, Molecular Function. p-value <0.05.

GO and KEGG analyses were performed to investigate the DEmRNA functions in IUA pathogenesis. GO analysis highlighted enrichment in extracellular matrix (ECM)-related terms, including “extracellular matrix structure,” “collagen-containing extracellular matrix,” and “collagen fibril organization” (Figures 2A,B; Supplementary Table S4). KEGG analysis revealed pathways associated with NF-κB signaling, apoptosis, Toll-like receptor signaling, and Notch signaling (Figures 2C,D; Supplementary Table S5).

### 3.3 Design and construction of the circRNA-miRNA-mRNA (ceRNA) network

**FIGURE 3:**
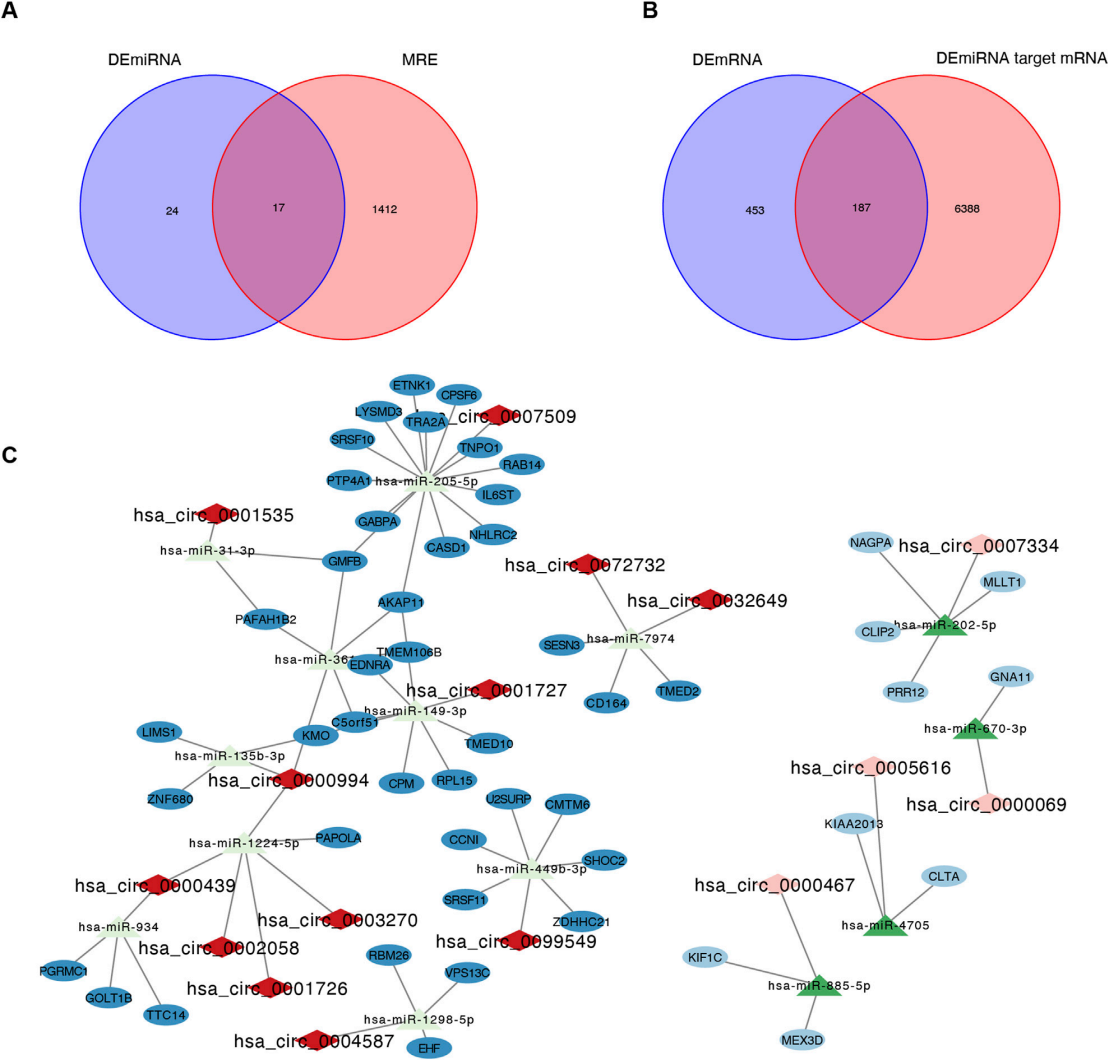
Identification of the circRNA-miRNA-mRNA regulatory network. **(A)** Intersection Venn diagram of differentially expressed miRNA (DEmiRNA) and MRE, yielding a total of 17 common miRNAs. **(B)** Intersection Venn diagram of differentially expressed mRNA (DEmRNA) and DEmiRNA target mRNA, with a total of 187 common mRNAs. **(C)** The circRNA-miRNA-mRNA regulatory network. The rhombus, triangular row and ellipse represent circRNAs, miRNAs, and mRNAs, respectively. Darker colors represent upregulated RNAs, and lighter colors represent downregulated RNAs. MRE, microRNA response element; IUA, intrauterine adhesion; NC, normal control.

A circRNA-miRNA-mRNA network was constructed based on the ceRNA hypothesis. By intersecting the 1,429 DEcircRNA-targeting miRNAs (MREs) with the 41 DEmiRNAs, we identified 17 common miRNAs (Figure 3A). Similarly, 6,575 target mRNAs were identified for the 41 DEmiRNAs, of which 128 common mRNAs overlapped with the previously identified 640 DEmRNAs (Figure 3B). Using these 44 DEcircRNAs, 17 common miRNAs, and 128 common mRNAs, a ceRNA network was visualized in Cytoscape. The final network consisted of 79 nodes (16 circRNAs, 14 miRNAs, and 49 mRNAs) and 75 edges (Figure 3C). Hub analysis using cytoHubba identified the top 5 circRNAs (hsa_circ_0000994, hsa_circ_0000439, hsa_circ_0000467, hsa_circ_0003270, and hsa-miR-670-3p) and the top 5 miRNAs (hsa-miR-205-5p, hsa-miR-149-3p, hsa-miR-449b-3p, hsa-miR-1224-5p, and hsa-miR-3614-5p) (Supplementary Table S6).

### 3.4 Differential expression of ceRNAs in IUA

**FIGURE 4:**
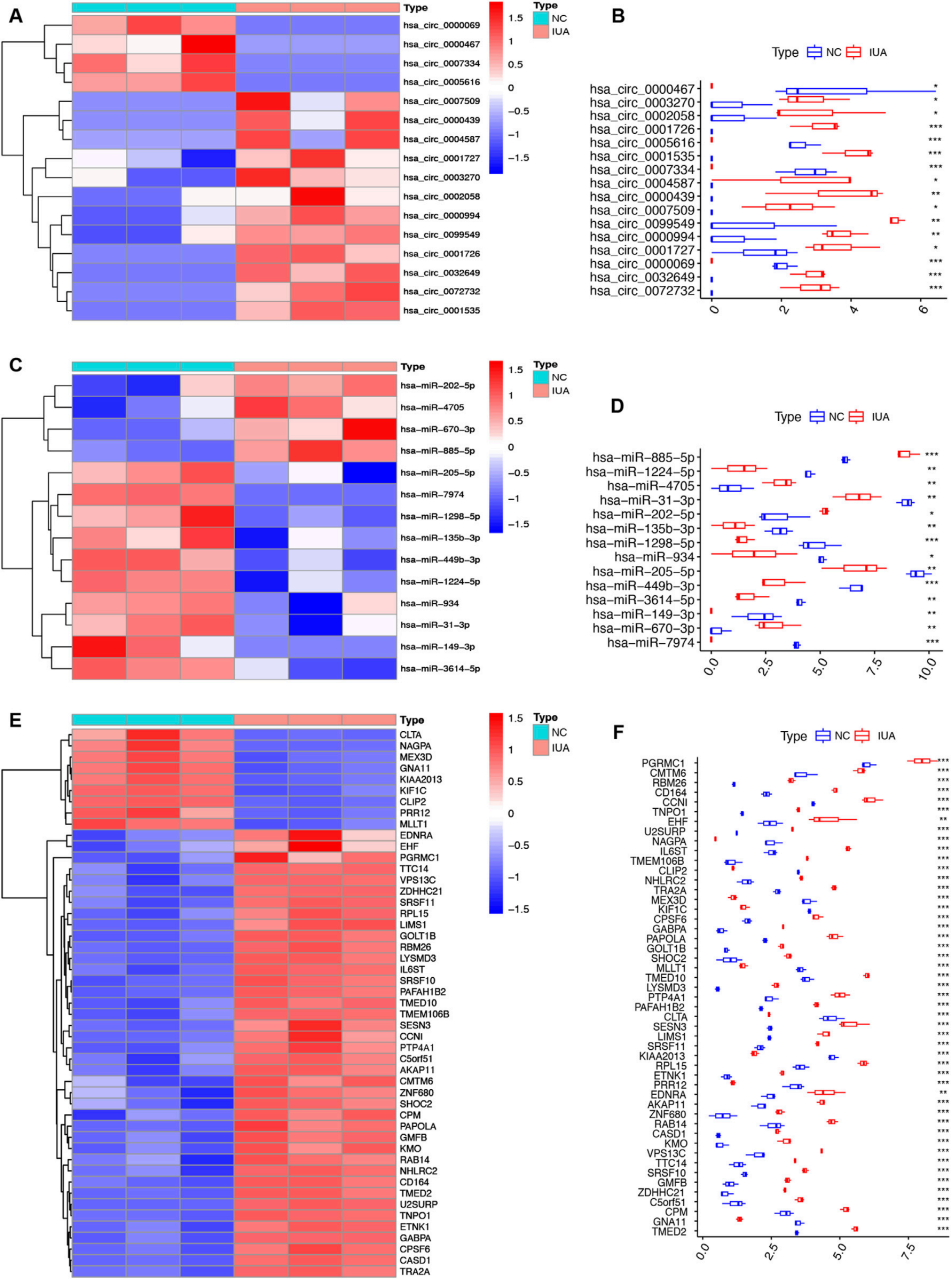
Heatmap and box plot of DEcircRNA **(A,B)**, DEmiRNA **(C,D)**, DEmRNA **(E,F)** in the circRNA-miRNA-mRNA regulatory network. IUA, intrauterine adhesion; NC, normal control. *p < 0.05; **p < 0.01; ***p < 0.001.

Heatmaps and box plots showed significant expression differences for the 16 circRNAs (Figures 4A,B), 14 miRNAs (Figures 4C,D), and 49 mRNAs (Figures 4E,F) between IUA and NC groups, suggesting their potential involvement in IUA pathogenesis.

### 3.5 Protein-protein interaction analysis and hub gene characterization

**FIGURE 5:**
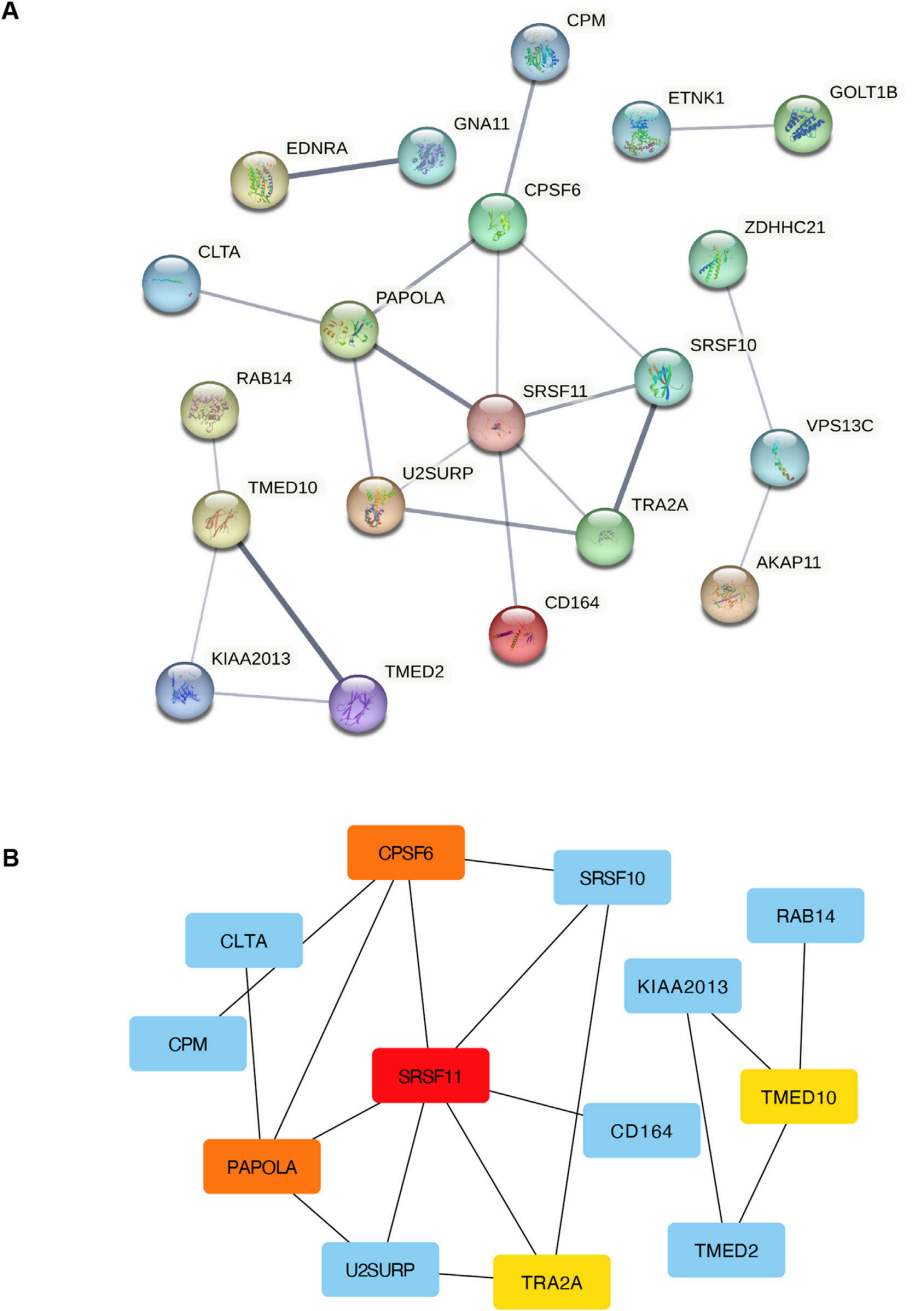
PPI and network analysis of DEmRNAs in the circRNA-miRNA-mRNA regulatory network. **(A)** PPI network of DEmRNAs in the circRNA-miRNA-mRNA regulatory network. **(B)** The network diagram of the top 10 hub mRNAs (colored) in the circRNA-miRNA-mRNA regulatory network. PPI, protein-protein interaction; DEmRNAs, differentially expressed miRNA-targeted- mRNAs.

PPI analysis of the 49 mRNAs in the ceRNA network was performed using STRING and visualized in Cytoscape. The resulting network included 20 nodes and 22 edges after excluding disconnected nodes and applying a medium-confidence threshold (Figure 5A). Hub gene analysis using cytoHubba identified the top 5 genes with the highest degrees of connectivity: SRSF11, PAPOLA, CPSF6, TRA2A, and TMED10 (Figure 5B).

### 3.6 Identification and validation of top two hub circRNAs in the ceRNAs network

**FIGURE 6:**
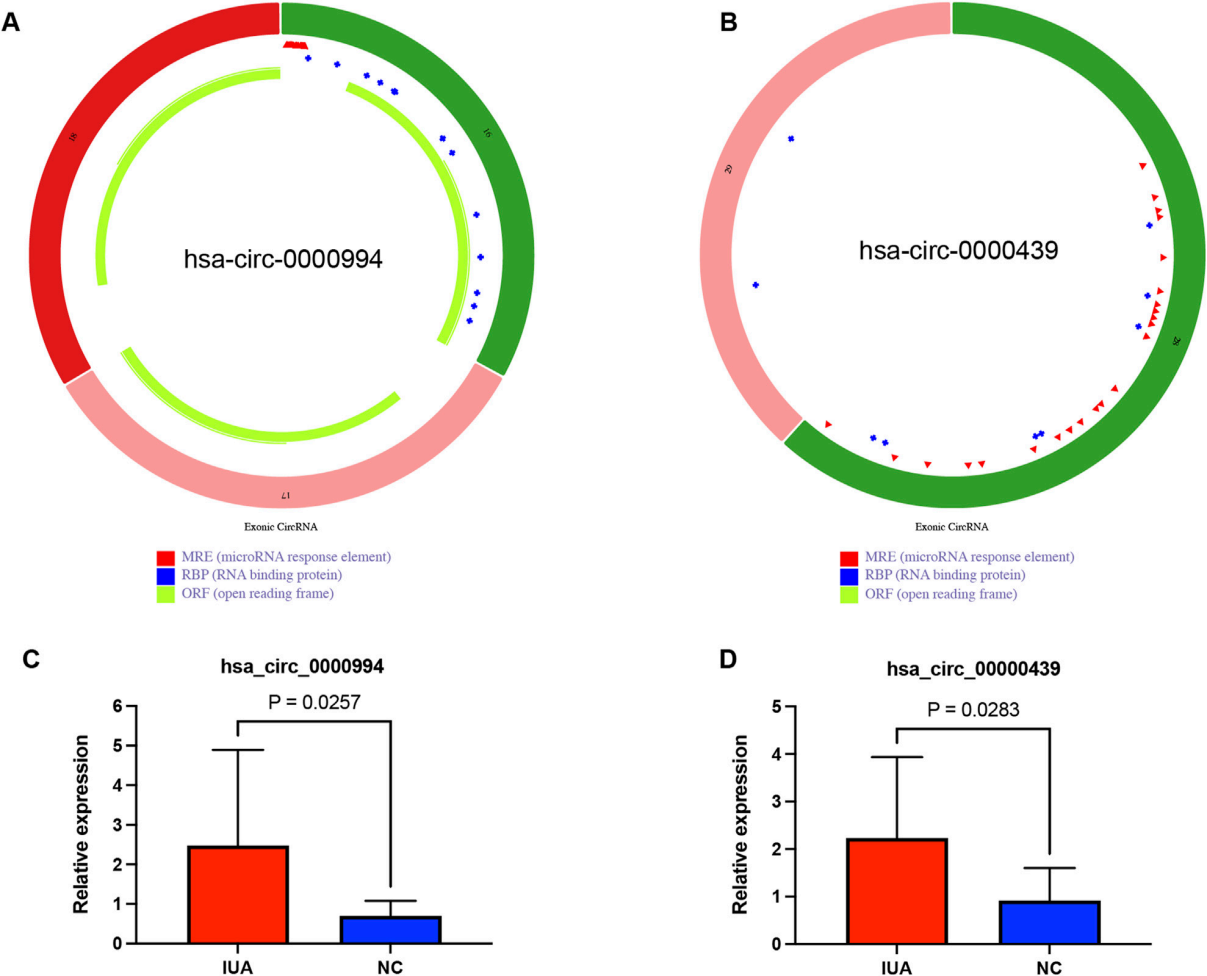
External clinical samples verified the differential expression level of two hub circRNAs. **(A)** Structure diagram of has_circ_0000994. **(B)** Structure diagram of has_circ_0000439. **(C)** The expression level of has_circ_0000994 in IUA (n = 11) and NC (n = 11) tissues was examined via qRT-PCR. **(D)** The expression level of has_circ_0000439 in IUA (n = 11) and NC (n = 11) tissues was examined via qRT-PCR.IUA, intrauterine adhesion; NC, normal control.

The top two hub circRNAs in the ceRNAs network were hsa_circ_0000994 and hsa_circ_0000439 based on degree scores in cytoHubba (Supplementary Table S6). The structures of these circRNAs are illustrated in Figures 6A,B. To validate their differential expression, RT-qPCR was performed using endometrial tissues from 11 IUA patients and 11 NC. Consistent with the RNA sequencing results, RT-qPCR revealed significantly higher expression levels of hsa_circ_0000994 and hsa_circ_0000439 in the IUA groups compared to the NC group (*p* < 0.05, Figures 6C,D).

### 3.7 Silencing of hsa_circ_0000994 inhibits TGF-β1-induced fibrosis markers in HESCs

**FIGURE 7:**
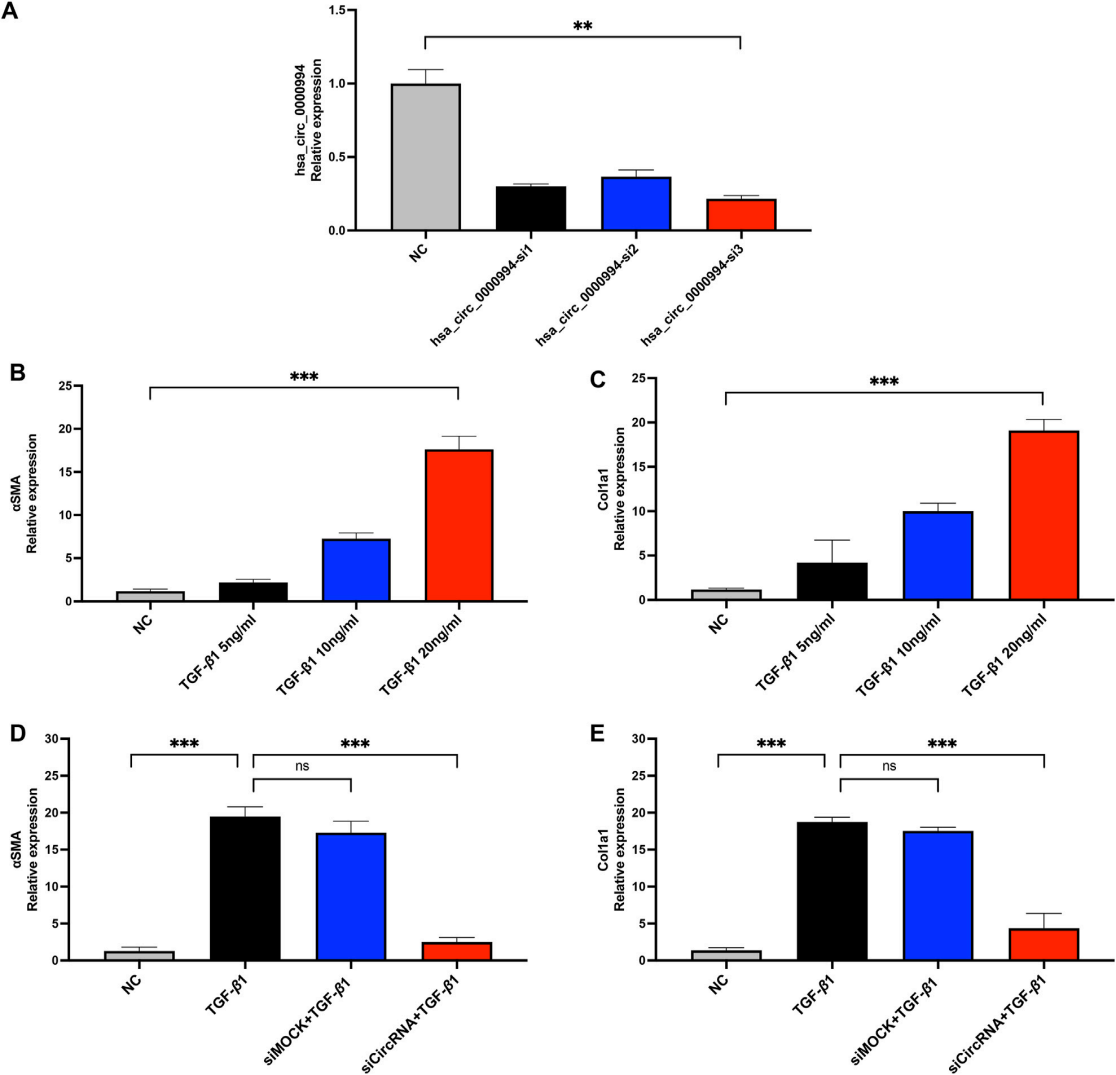
Knockdown of hsa-circ-0000994 attenuates fibrosis *in vitro*. **(A)** qPCR detection of hsa-circ-0000994 expression in HESCs cells in diffrent transfection group. **(B,C)** Expression levels of α-SMA and COL1A1 in HESCs cells treated with different concentrations of TGF-β1. **(D,E)** Expression levels of α-SMA and COL1A1 in HESCs cells treated with siCircRNA. siCircRNA:hsa-circ-0000994 small interfering RNA 3 treated group; NC, normal control group.

To assess the knockdown efficiency of the most differentially significant cicRNA hsa_circ_0000994, siRNA transfection were performed. Among the three siRNA candidates, hsa_circ_0000994-si3 showed the highest knockdown efficiency and was selected for subsequent experiments (Figure 7A). Fibrosis markers α-SMA and COL1A1 were markedly upregulated following TGF-β1 exposure, with the 20 ng/mL group showing the most pronounced increase (*p* < 0.05, Figures 7B,C). Thus, 20 ng/mL TGF-β1was chosen for further experiments. In the siCircRNA-mediated silencing of hsa_circ_0000994 group, a significant decrease in α-SMA and COL1A1 expression was observed relative to the levels in the TGF-β1 group. (*p* < 0.05, Figures 7D,E). The findings indicate that hsa_circ_0000994 could be an important regulatory factor involved in fibrosis associated with IUA.

## 4 Discussion

Although circRNAs have been implicated in fibrotic diseases such as cardiac and liver fibrosis (Zhou et al., 2018), their role in the pathogenesis of IUA remains poorly understood. This study addressed this gap by performing circRNA sequencing and comprehensive bioinformatics analysis, revealing significant differences in circRNA, miRNA, and mRNA expression between IUA and normal endometrial tissues. Moreover, we constructed a circRNA-miRNA-mRNA ceRNA network, providing novel insights into the molecular mechanisms underlying IUA.

CircRNAs play diverse roles as miRNA sponges, protein-binding molecules, and transcriptional regulators, often contributing to ceRNA networks (Cheng et al., 2019). For instance, overexpression of circPlekha7 has been reported to suppress α-SMA, type I collagen, and Smad3 expression, thereby modulating endometrial epithelial cell activity and promoting apoptosis (Wei et al., 2020). In our study, compared to normal endometrial tissues, we identified 44 differentially expressed circRNAs in IUA endometrium, including 13 upregulated and 31 downregulated circRNAs. Notably, hsa_circ_0000994 and hsa_circ_0000439 emerged as the top two hub circRNAs in the ceRNA network. Hsa_circ_0000994, also known as circSLC8A1, is located in the SLC8A1 gene region. Prior studies have demonstrated its dysregulation in various diseases, including bladder cancer (Lu et al., 2019), Parkinson’s disease (Hanan et al., 2020), and renal fibrosis (Wei et al., 2022). Wei et al. found that hsa_circ_0000994 was dysregulated in the unilateral ischemia-reperfusion injury (UIRI) model and may play an integral part in the pathogenesis of renal fibrosis, indicating that these aberrantly expressed circRNAs could serve as biomarkers for early diagnosis and therapeutic intervention to prevent disease progression (Wei et al., 2022). The hsa_circ_0000439 also has been found upregulated in gastric cancer and adjacent non-tumor tissues (Vidal et al., 2017). In our study, hsa_circ_0000994 was significantly upregulated in the IUA group based on sequencing results, and the qPCR results were consistent with the sequencing data. siRNA-mediated knockdown of hsa_circ_0000994 in the IUA cell model also resulted in reduced expression of fibrosis markers, suggesting that hsa_circ_0000994 may play an important role in the regulation of IUA fibrosis.

Further analysis revealed potential downstream targets of the hub circRNAs. LIMS1, located downstream of hsa_circ_0000994, encodes a LIM domain-containing protein and is a known translocation partner in lipoma (Sporkova et al., 2023). Another downstream target, KMO, is a key enzyme in the kynurenine pathway, where its end product, NAD+, is critical for ATP production, mitochondrial dynamics, and ROS regulation (Castro-Portuguez and Sutphin, 2020). Dysregulated KMO metabolism has been linked to oxidative stress and mitochondrial dysfunction, both of which are relevant to endometrial fibrosis (Atkins et al., 2019). For hsa_circ_0000439, one significant downstream gene is PGRMC1, which encodes progesterone receptor membrane component 1. PGRMC1 is implicated in hormone sensitivity and cholesterol synthesis regulation via the Akt signaling pathway and has been extensively studied in endometrial and cervical tumors (McGuire and Espenshade, 2023). These findings suggest that both hub circRNAs may regulate fibrosis and inflammation-related pathways in IUA, highlighting their potential as therapeutic targets.

GO and KEGG enrichment analyses of DEmRNAs further elucidated the molecular mechanisms of IUA. DEmRNAs were significantly enriched in ECM-related terms. KEGG analysis identified key pathways, including NF-κB signaling, Notch signaling, as critical to IUA pathogenesis. The NF-κB signaling pathway is a well-established regulator of inflammation, immune responses, and cell proliferation. Its activation involves phosphorylation of IκB by the IκB kinase complex, which facilitates NF-κB nuclear translocation and transcriptional regulation of target genes (Haga and Okada, 2022; Ma and Hottiger, 2016). Studies have demonstrated elevated phosphorylated IκB levels in IUA tissues, suggesting hyperactivation of NF-κB signaling in the formation of intrauterine adhesions (Trackman et al., 2015). Moreover, NF-κB promotes TGF-β1 expression while suppressing MMP-9, contributing to ECM deposition and fibrosis in IUA (Rial et al., 2012). The Notch signaling pathway, known for its role in fibrotic processes across multiple organs, has also been implicated in IUA. Xu et al. demonstrated increased Notch receptor expression and collagen deposition in a murine IUA model, while treatment with the γ-secretase inhibitor DAPT alleviated fibrosis (Sprinzak and Blacklow, 2021; Xu et al., 2021). These findings suggest that targeting the NF-κB and Notch pathways may hold therapeutic potential for managing IUA.

Although the study yielded these significant results, it is subject to certain limitations. First, the relatively small sample size for RNA sequencing may restrict the generalizability of the results, and larger cohorts are needed to validate the identified biomarkers. Second, although the differential expression and role of hub circRNAs was confirmed by RT-qPCR and siRNA knockdown, additional functional validation experiments are essential to confirm their direct molecular interactions and regulatory roles in IUA pathogenesis, such as RNA immunoprecipitation or luciferase assays. Future studies should aim to validate these findings in a larger, multi-center, and ethnically diverse cohort, ideally incorporating longitudinal sampling to monitor dynamic changes in circRNA expression during fibrosis progression or treatment.

In conclusion, this study presents a comprehensive analysis of dysregulated circRNAs, miRNAs, and mRNAs in the endometrial tissues of IUA patients, leading to the construction of a circRNA–miRNA–mRNA regulatory network. Notably, hsa_circ_0000994 was identified and experimentally validated as a key circRNA potentially involved in the regulation of fibrosis in IUA, thereby expanding upon previous bioinformatics-based findings. By identifying key circRNAs and their associated pathways, we provide novel molecular insights and lay the groundwork for the development of targeted therapeutic strategies for IUA. These findings not only enhance our understanding of IUA pathogenesis but also suggest that hsa_circ_0000994 may serve as a potential diagnostic biomarker or therapeutic target, laying the groundwork for the development of novel anti-fibrotic strategies in reproductive medicine.

## Data Availability

The data presented in the study are deposited in the GSA for Human repository, accession number HRA012470.
