# Supplementary material for: Identification and experimental validation of circular RNA-associated ceRNA networks in intrauterine adhesion
Source: Front Genet. 2025 Aug 29;16:1619698. doi: 10.3389/fgene.2025.1619698 (PMC12426003; doi:10.3389/fgene.2025.1619698)
Supplement: Supplementary file 1 [file Supplementaryfile1.docx]

**Supplemental Figures**


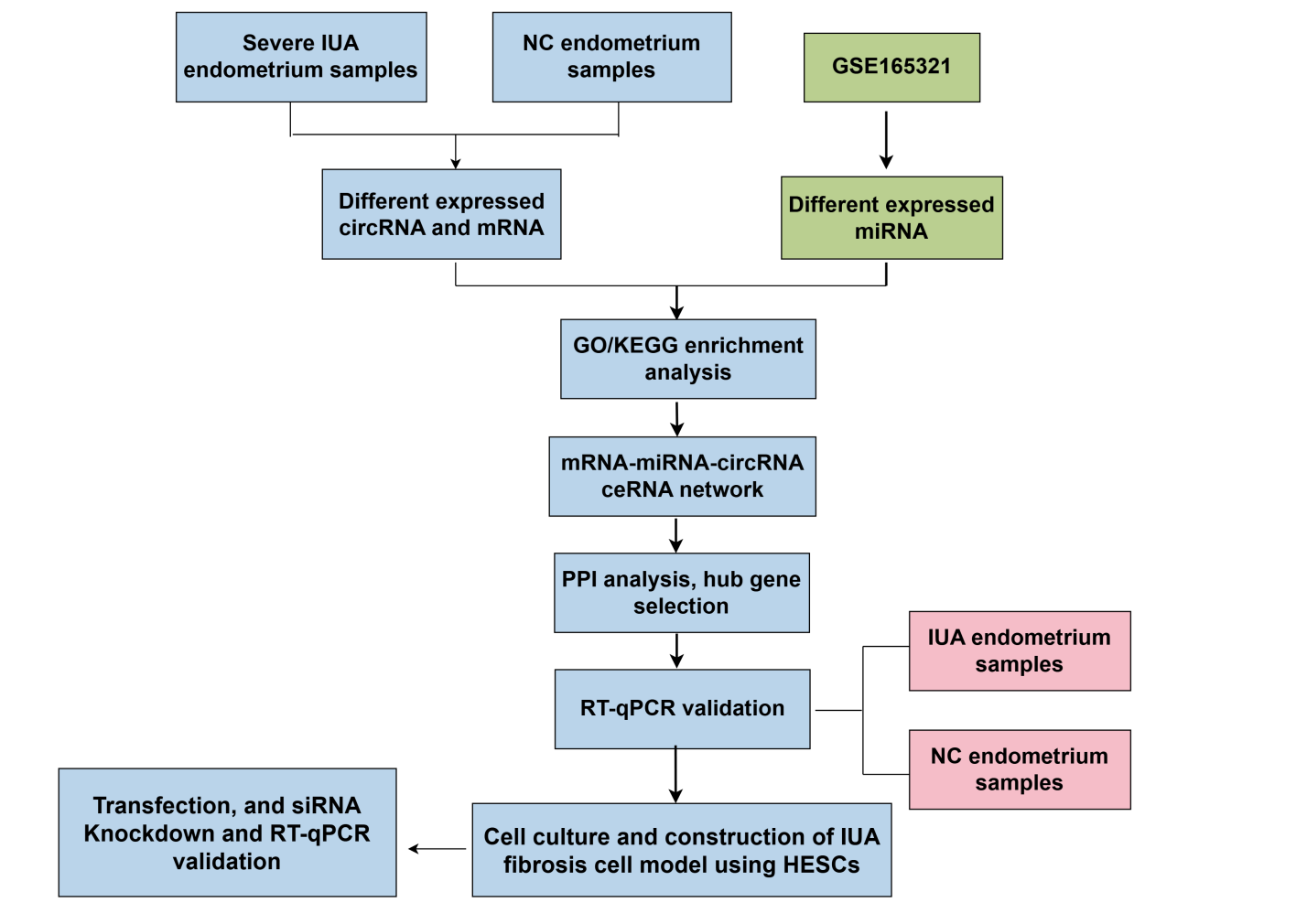


**Supplemental Figure 1.** Graphical abstract of this study.


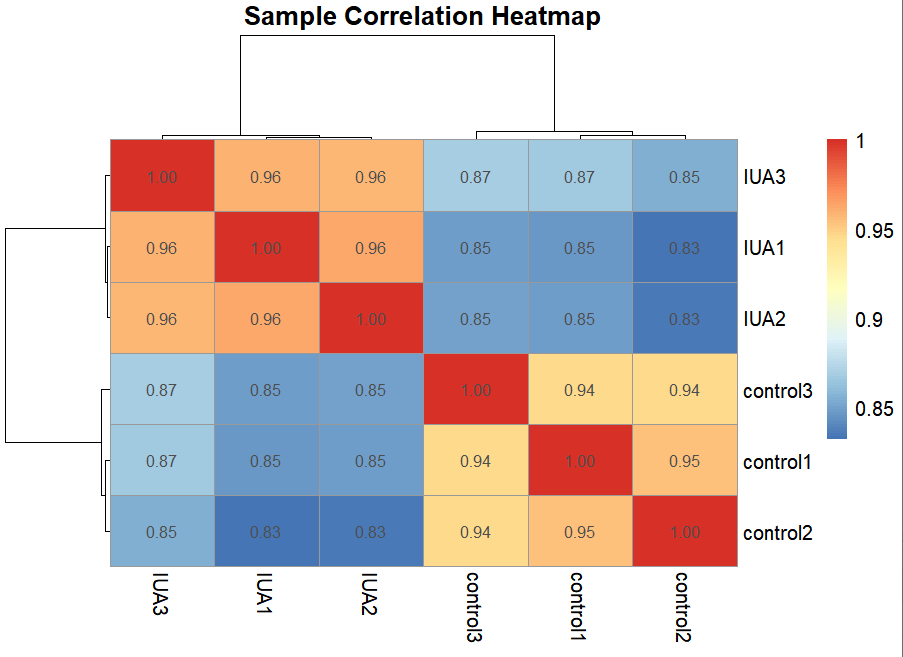


**Supplementary Figure 2：**Sample correlation heatmap of RNA-seq transcriptomic data.

Each cell represents the Pearson correlation coefficient of transcriptomic expression profiles (FPKM values) between a pair of samples. Samples from the IUA group (IUA 1–3) show high intra-group correlation (r ≥ 0.94), as do the control group samples (control 1–3). In contrast, cross-group correlations (IUA vs. control) are markedly lower (r ≤ 0.87), indicating strong within-group consistency and good separation between groups.

**Supplementary Tables**

**Supplementary Table 1.** Differentially expressed circRNAs between intrauterine adhesion and normal control.

| **ID** | **logFC** | **AveExpr** | **t** | **p-value** |
| --- | --- | --- | --- | --- |
| *hsa_circ_0001535* | 4.099318187 | 2.049659094 | 9.867138907 | 3.29E-05 |
| *hsa_circ_0016601* | 4.213739155 | 2.106869578 | 8.940015503 | 6.09E-05 |
| *hsa_circ_0032649* | 2.892576084 | 1.446288042 | 8.924001886 | 6.16E-05 |
| *hsa_circ_0000095* | -3.002401402 | 1.788418757 | -8.689065544 | 7.26E-05 |
| *hsa_circ_0005616* | -2.544741979 | 1.27237099 | -8.253212913 | 9.96E-05 |
| *hsa_circ_0001726* | 3.148252359 | 1.574126179 | 7.752067634 | 0.000145901 |
| *hsa_circ_0000069* | -2.023356541 | 1.01167827 | -7.511418805 | 0.000176508 |
| *hsa_circ_0001023* | 3.969151939 | 1.98457597 | 7.389927106 | 0.00019469 |
| *hsa_circ_0105527* | 3.421364286 | 1.710682143 | 6.774256409 | 0.000326721 |
| *hsa_circ_0103136* | 3.374295373 | 1.687147686 | 6.635826088 | 0.00036892 |
| *hsa_circ_0072732* | 2.920937501 | 1.460468751 | 6.63276921 | 0.000369919 |
| *hsa_circ_0007334* | -2.794831253 | 1.397415627 | -6.216326657 | 0.000539652 |
| *hsa_circ_0005603* | 2.629833874 | 1.314916937 | 6.071019076 | 0.000618412 |
| *hsa_circ_0005615* | 2.345308801 | 2.983677513 | 5.629876922 | 0.000949361 |
| *hsa_circ_0001522* | 3.620751374 | 2.559923081 | 5.52832344 | 0.001051324 |
| *hsa_circ_0007883* | 2.838237739 | 1.41911887 | 5.492215422 | 0.001090499 |
| *hsa_circ_0003611* | 3.567703096 | 1.783851548 | 5.193848296 | 0.001484919 |
| *hsa_circ_0000994* | 3.091162254 | 2.166132516 | 5.070132079 | 0.001693532 |
| *hsa_circ_0000024* | -2.4307494 | 1.566592928 | -4.803409228 | 0.002264491 |
| *hsa_circ_0004771* | 3.037796982 | 3.032814754 | 4.655316233 | 0.002672397 |
| *hsa_circ_0000373* | 2.583094051 | 1.291547025 | 4.340952416 | 0.003838508 |
| *hsa_circ_0072688* | 3.287830469 | 2.253764119 | 4.289374976 | 0.004079142 |
| *hsa_circ_0000439* | 3.680804114 | 1.840402057 | 4.282281823 | 0.004113522 |
| *hsa_circ_0099549* | 4.079191518 | 3.236185844 | 4.261598491 | 0.004215617 |
| *hsa_circ_0003423* | 3.109927068 | 2.135586374 | 4.138529726 | 0.004884168 |
| *hsa_circ_0002484* | 3.136248077 | 3.01085774 | 4.062752232 | 0.005353616 |
| *hsa_circ_0072547* | -2.642202257 | 1.321101128 | -3.796128723 | 0.00744541 |
| *hsa_circ_0008967* | 2.000265756 | 2.201307107 | 3.647577527 | 0.008989345 |
| *hsa_circ_0082096* | 2.651206053 | 1.325603026 | 3.563281366 | 0.010018688 |
| *hsa_circ_0083444* | 2.329988743 | 1.164994371 | 3.518294725 | 0.010620053 |
| *hsa_circ_0007509* | 2.21488204 | 1.10744102 | 3.497920955 | 0.010905232 |
| *hsa_circ_0003270* | 2.202935499 | 1.682090589 | 3.220307949 | 0.015742404 |
| *hsa_circ_0000992* | 2.060570507 | 1.030285254 | 3.20968547 | 0.015968598 |
| *hsa_circ_0000467* | -3.576960541 | 1.78848027 | -3.146538873 | 0.017387554 |
| *hsa_circ_0135288* | 3.123847383 | 1.561923692 | 3.006461659 | 0.021042087 |
| *hsa_circ_0039353* | 2.150766151 | 1.695934465 | 2.985559533 | 0.021654469 |
| *hsa_circ_0016600* | 2.557138651 | 1.278569326 | 2.892041297 | 0.024636665 |
| *hsa_circ_0001727* | 2.133545872 | 2.498804133 | 2.697962562 | 0.032307452 |
| *hsa_circ_0011422* | 2.232556008 | 1.116278004 | 2.573302138 | 0.038532707 |
| *hsa_circ_0003632* | 3.137810548 | 1.568905274 | 2.533979384 | 0.040747343 |
| *hsa_circ_0004587* | 2.654138485 | 1.327069243 | 2.527221039 | 0.041141141 |
| *hsa_circ_0001519* | 2.528970027 | 1.845107853 | 2.521264909 | 0.04149148 |
| *hsa_circ_0000139* | 2.366481698 | 1.183240849 | 2.467692026 | 0.044785601 |
| *hsa_circ_0002058* | 2.310426033 | 1.775764406 | 2.413793072 | 0.048374338 |

The filtering criteria of p-value < 0.05 and | log_2_ fold change (FC)| > 2.

**Supplementary Table 2.** Differentially expressed miRNAs between intrauterine adhesion and normal control.

| **ID** | **logFC** | **AveExpr** | **t** | **p-value** |
| --- | --- | --- | --- | --- |
| *hsa-miR-133a-3p* | 6.578692998 | 10.35459853 | 19.04312293 | 3.87E-07 |
| *hsa-miR-1-3p* | 6.632341311 | 11.15607986 | 18.28484906 | 5.07E-07 |
| *hsa-miR-7974* | -3.914036643 | 1.957018322 | -15.83268087 | 1.32E-06 |
| *hsa-miR-1-5p* | 4.866572931 | 3.10939341 | 15.39373652 | 1.59E-06 |
| *hsa-miR-133a-5p* | 6.704417158 | 4.96341014 | 14.75890038 | 2.10E-06 |
| *hsa-miR-133b* | 7.086712538 | 6.503150452 | 13.08016249 | 4.64E-06 |
| *hsa-miR-143-3p* | 2.225023144 | 19.28495013 | 9.271077274 | 4.29E-05 |
| *hsa-miR-383-5p* | 5.413688562 | 3.776107648 | 8.206927086 | 9.22E-05 |
| *hsa-miR-885-5p* | 2.771403655 | 7.539237985 | 7.756215758 | 0.000130916 |
| *hsa-miR-145-5p* | 2.365724955 | 15.34345614 | 7.589254481 | 0.000149689 |
| *hsa-miR-5002-5p* | 2.581474809 | 1.401848516 | 6.940082766 | 0.000258096 |
| *hsa-miR-605-3p* | 2.504411631 | 3.379242835 | 6.414155223 | 0.000413565 |
| *hsa-miR-449a* | -2.575140649 | 12.59620333 | -6.200271798 | 0.000505206 |
| *hsa-miR-1298-5p* | -3.36597874 | 3.135611866 | -6.053793686 | 0.000581147 |
| *hsa-miR-490-3p* | 3.232487754 | 4.175695155 | 5.768213763 | 0.000769108 |
| *hsa-miR-449b-3p* | -3.547173574 | 4.79575386 | -5.764899259 | 0.000771658 |
| *hsa-miR-203a-5p* | -2.389964591 | 1.616369101 | -5.619209531 | 0.000893688 |
| *hsa-miR-135b-5p* | -2.096862469 | 9.938892558 | -5.533728592 | 0.000975285 |
| *hsa-miR-548ax* | 2.4181115 | 1.824434394 | 5.294887905 | 0.001251147 |
| *hsa-miR-3614-5p* | -2.399555989 | 2.874831164 | -5.227203351 | 0.001344477 |
| *hsa-miR-551b-3p* | 2.016776212 | 3.210915011 | 5.196034562 | 0.001390052 |
| *hsa-miR-1224-5p* | -3.126048887 | 2.922777737 | -4.978646797 | 0.001760327 |
| *hsa-miR-449c-5p* | -2.393753633 | 10.65124418 | -4.839796323 | 0.002053923 |
| *hsa-miR-885-3p* | 2.204753634 | 2.496528901 | 4.342405704 | 0.003650912 |
| *hsa-miR-449c-3p* | -4.26428951 | 5.301250596 | -4.335699635 | 0.003680249 |
| *hsa-miR-1283* | -2.510058106 | 1.908078359 | -4.26600693 | 0.004001033 |
| *hsa-miR-670-3p* | 2.534622872 | 1.57758713 | 4.206575778 | 0.00429905 |
| *hsa-miR-124-5p* | -2.73836476 | 1.36918238 | -4.137621911 | 0.004675855 |
| *hsa-miR-196a-5p* | 2.780883967 | 7.359319824 | 4.075904811 | 0.005044104 |
| *hsa-miR-548ba* | 2.794911372 | 2.132390058 | 3.902979189 | 0.006256964 |
| *hsa-miR-149-3p* | -2.204322532 | 1.102161266 | -3.867995718 | 0.006539345 |
| *hsa-miR-31-3p* | -2.231205716 | 7.840152573 | -3.833178451 | 0.006834293 |
| *hsa-miR-4705* | 2.313543629 | 2.064541259 | 3.786099977 | 0.007256491 |
| *hsa-miR-135b-3p* | -2.090757577 | 2.076614479 | -3.612451455 | 0.009078258 |
| *hsa-miR-205-5p* | -2.737570496 | 8.117232183 | -3.57952061 | 0.009477004 |
| *hsa-miR-202-5p* | 2.147801002 | 4.139883956 | 3.430586086 | 0.011533767 |
| *hsa-miR-934* | -3.102282481 | 3.52051 | -3.405854619 | 0.011919887 |
| *hsa-miR-519a-3p* | -2.138250615 | 2.849908392 | -3.07052997 | 0.018787334 |
| *hsa-miR-124-3p* | -3.449575159 | 6.274782885 | -2.784617144 | 0.028013203 |
| *hsa-miR-875-5p* | -2.818067301 | 2.725353104 | -2.646174387 | 0.034104033 |
| *hsa-miR-3117-3p* | -2.893987468 | 3.870255644 | -2.637946237 | 0.034507206 |

The filtering criteria of p-value < 0.05 and |log_2_ fold change (FC)| > 2.

**Supplementary Table 3:** Differentially expressed mRNAs between intrauterine adhesion and normal control.

| **ID** | **logFC** | **AveExpr** | **t** | **p-value** |
| --- | --- | --- | --- | --- |
| *TIA1* | 2.815437607 | 3.451787921 | 39.26558783 | 0.000161992 |
| *MMP1* | -3.451094614 | 1.739588682 | -38.7067622 | 0.000161992 |
| *MMP3* | -4.832223489 | 2.45504963 | -38.10228261 | 0.000161992 |
| *PMVK* | -3.035944156 | 2.873895318 | -38.10087812 | 0.000161992 |
| *SNX8* | -2.571354787 | 1.966840821 | -37.84487593 | 0.000161992 |
| *CLIP2* | -2.388851845 | 2.301551601 | -35.40208729 | 0.000161992 |
| *SLC9A3R2* | -3.023251074 | 2.947610689 | -35.20200593 | 0.000161992 |
| *HDGFL2* | -3.480268171 | 2.785259222 | -33.70081558 | 0.000161992 |
| *SURF6* | -2.891706748 | 2.602249106 | -33.13487633 | 0.000161992 |
| *U2SURP* | 2.043616691 | 2.254457264 | 33.01502244 | 0.000161992 |
| *DGKQ* | -2.595894919 | 2.057051119 | -32.72573385 | 0.000161992 |
| *CAVIN1* | -2.820209281 | 3.907217131 | -31.94873308 | 0.000161992 |
| *CXCL8* | -4.884356619 | 2.637598387 | -30.65655835 | 0.000161992 |
| *CHTF18* | -2.5375699 | 2.018591957 | -30.55199081 | 0.000161992 |
| *PIK3C2A* | 2.255495386 | 2.685207827 | 30.54883832 | 0.000161992 |
| *TOR4A* | -2.712977784 | 2.062277322 | -29.91724866 | 0.000161992 |
| *SART1* | -2.765430292 | 2.097344138 | -29.75860969 | 0.000161992 |
| *RRBP1* | -3.449284192 | 4.168338452 | -29.72641474 | 0.000161992 |
| *EHD2* | -2.042992273 | 3.695759868 | -29.6677359 | 0.000161992 |
| *APOE* | -3.298690741 | 2.245126918 | -28.92972241 | 0.000161992 |
| *INAFM1* | -2.978385234 | 2.131522126 | -28.76585643 | 0.000161992 |
| *BIN1* | -2.403774336 | 2.509404571 | -28.58048965 | 0.000161992 |
| *RCN3* | -2.764376052 | 2.763238533 | -28.56002412 | 0.000161992 |
| *TNPO1* | 2.055517377 | 2.455745372 | 28.43936773 | 0.000161992 |
| *PALM* | -2.796837193 | 2.322442562 | -28.33838593 | 0.000161992 |
| *FOXO6* | -2.263331889 | 1.181093079 | -27.86593989 | 0.000161992 |
| *PHLDB3* | -2.126986669 | 1.648216193 | -27.79501688 | 0.000161992 |
| *ATP5F1D* | -3.521263037 | 2.940740088 | -27.63251211 | 0.000161992 |
| *ATAD3B* | -2.010438028 | 1.649162731 | -27.62014374 | 0.000161992 |
| *MAZ* | -2.527809277 | 2.768535168 | -27.50136739 | 0.000161992 |
| *C6orf47* | -2.825745829 | 2.371443163 | -26.8268651 | 0.000161992 |
| *CFAP410* | -2.160857592 | 2.147542964 | -26.709235 | 0.000161992 |
| *TM7SF2* | -2.328816102 | 2.44386796 | -26.68844714 | 0.000161992 |
| *KIF26A* | -3.11694876 | 2.490866766 | -26.04102631 | 0.000161992 |
| *ARID5A* | -2.270145881 | 2.152661452 | -25.97089768 | 0.000161992 |
| *PLEC* | -3.394385423 | 3.413421836 | -25.7315708 | 0.000161992 |
| *KRI1* | -2.062638688 | 1.837725625 | -25.66332671 | 0.000161992 |
| *CROCC* | -2.885727237 | 2.327264159 | -25.65327561 | 0.000161992 |
| *BOP1* | -2.349948118 | 2.352347522 | -25.38366537 | 0.000161992 |
| *SPATA2L* | -2.777837056 | 1.766601602 | -25.31486023 | 0.000161992 |
| *C11orf95* | -2.462437169 | 2.897424757 | -25.2238805 | 0.000161992 |
| *MBD3* | -2.671166617 | 2.447112306 | -25.21482411 | 0.000161992 |
| *STMN3* | -2.855709265 | 2.46228277 | -25.18208579 | 0.000161992 |
| *CTXN1* | -5.022935964 | 3.075717106 | -25.0694484 | 0.000161992 |
| *NINL* | -2.191838077 | 2.21595638 | -25.00926563 | 0.000161992 |
| *SUN2* | -2.308206312 | 2.697767804 | -24.71041432 | 0.000161992 |
| *CCDC61* | -2.217919705 | 1.761492608 | -24.67552605 | 0.000161992 |
| *CASD1* | 2.161321465 | 1.649746876 | 24.59472393 | 0.000161992 |
| *DOHH* | -2.760382467 | 1.731037898 | -24.43157626 | 0.000161992 |
| *SDF4* | -3.203922011 | 3.340476308 | -24.3427474 | 0.000161992 |
| *EHBP1L1* | -2.451613836 | 2.6267594 | -24.33694914 | 0.000161992 |
| *BICDL2* | -2.60133226 | 1.950741859 | -24.33279262 | 0.000161992 |
| *GSDMD* | -2.722026258 | 2.405867722 | -24.30586524 | 0.000161992 |
| *NUDC* | -2.667446274 | 2.653212868 | -23.93046483 | 0.000161992 |
| *SCARF2* | -2.031361625 | 2.412358293 | -23.88317736 | 0.000161992 |
| *DMTF1* | 2.323915788 | 2.919116966 | 23.87341242 | 0.000161992 |
| *ETFB* | -2.171934999 | 1.705682107 | -23.86004078 | 0.000161992 |
| *NCOR2* | -2.2084259 | 2.981184665 | -23.67873229 | 0.000161992 |
| *COL5A1* | -2.781920232 | 5.664607672 | -23.59676788 | 0.000161992 |
| *PRDX5* | -3.21674925 | 3.107909519 | -23.52242649 | 0.000161992 |
| *NFKBIB* | -2.23146363 | 1.493134878 | -23.49940558 | 0.000161992 |
| *AKT1* | -2.031953117 | 2.352147185 | -23.45860964 | 0.000161992 |
| *MYDGF* | -2.573892601 | 2.297664566 | -23.42710546 | 0.000161992 |
| *LIG1* | -2.101756302 | 2.100097002 | -23.33686102 | 0.000161992 |
| *RRP1* | -2.461876081 | 2.075965429 | -23.31018645 | 0.000161992 |
| *SLC39A6* | 2.466172526 | 5.23780535 | 23.30139759 | 0.000161992 |
| *PLEKHH3* | -2.798159048 | 2.175665753 | -23.24635154 | 0.000161992 |
| *GFER* | -2.520520852 | 1.531857691 | -23.22912825 | 0.000161992 |
| *ZNF469* | -2.520251847 | 1.864042343 | -22.96905127 | 0.000161992 |
| *DPP7* | -2.363889822 | 2.529529287 | -22.81789304 | 0.000161992 |
| *TMED2* | 2.138907109 | 4.497381792 | 22.80345374 | 0.000161992 |
| *DVL1* | -2.336142276 | 2.437540997 | -22.69983786 | 0.000161992 |
| *NELFB* | -3.133234605 | 3.076318903 | -22.59980683 | 0.000161992 |
| *FARSA* | -2.033007391 | 3.61663375 | -22.59483731 | 0.000161992 |
| *HMG20B* | -2.883339283 | 2.668478262 | -22.59192332 | 0.000161992 |
| *PAFAH1B2* | 2.01836476 | 3.136509561 | 22.36360541 | 0.000161992 |
| *ZNF205* | -2.04925699 | 1.618719369 | -22.30681232 | 0.000161992 |
| *STK11* | -2.820381667 | 1.993064908 | -22.2624588 | 0.000161992 |
| *MBNL2* | 2.552786988 | 3.029104939 | 22.22193595 | 0.000161992 |
| *PPP1R9B* | -2.74724974 | 3.04268455 | -22.19284485 | 0.000161992 |
| *PFKL* | -2.716203787 | 3.013813921 | -21.95091153 | 0.000161992 |
| *PGR* | 2.596458449 | 5.198660199 | 21.95077514 | 0.000161992 |
| *NCAPH2* | -2.761202572 | 2.628992015 | -21.88899672 | 0.000161992 |
| *RBM26* | 2.098789292 | 2.178323778 | 21.80538204 | 0.000161992 |
| *CEP170B* | -2.292105983 | 2.625650347 | -21.78275826 | 0.000161992 |
| *ADAM8* | -2.117631359 | 1.407826016 | -21.64660236 | 0.000161992 |
| *D2HGDH* | -2.305840016 | 2.263062295 | -21.61912092 | 0.000161992 |
| *MTPN* | 2.221291157 | 4.663976219 | 21.52254334 | 0.000161992 |
| *ZBTB7A* | -2.450069834 | 2.446389836 | -21.48663598 | 0.000161992 |
| *SCAF1* | -3.592614152 | 3.084752083 | -21.42262839 | 0.000161992 |
| *E4F1* | -2.254604958 | 2.056999525 | -21.40436537 | 0.000161992 |
| *GOLT1B* | 2.013685152 | 1.866570662 | 21.22406855 | 0.000161992 |
| *NIBAN2* | -2.992028342 | 3.783451506 | -21.19706661 | 0.000161992 |
| *CTBP1* | -2.360181583 | 3.188471462 | -21.17099746 | 0.000161992 |
| *SRSF10* | 2.19847078 | 2.627858568 | 21.1577807 | 0.000161992 |
| *ZNF316* | -3.182770013 | 2.505094812 | -21.10098371 | 0.000161992 |
| *MMP10* | -3.848345729 | 2.091460503 | -21.08228501 | 0.000161992 |
| *CHAF1A* | -2.072776491 | 2.176381042 | -21.0768921 | 0.000161992 |
| *NUBP2* | -2.169882639 | 2.096588456 | -21.06464896 | 0.000161992 |
| *LYSMD3* | 2.130178464 | 1.607100989 | 20.98371331 | 0.00016212 |
| *TRIR* | -2.806683405 | 3.311330839 | -20.90774117 | 0.00016212 |
| *HELZ2* | -2.465787196 | 1.988569787 | -20.88776884 | 0.00016212 |
| *FDXR* | -2.512726027 | 1.764893154 | -20.82969651 | 0.000163427 |
| *SMARCAD1* | 2.320828162 | 2.122211231 | 20.81131053 | 0.000163427 |
| *SF3B1* | 2.163110754 | 4.258313019 | 20.71175535 | 0.000163714 |
| *ZRANB2* | 2.701307768 | 2.741736173 | 20.6193837 | 0.000164196 |
| *SCRIB* | -3.468747896 | 2.880190973 | -20.60095986 | 0.000164196 |
| *WDR90* | -2.079854694 | 1.973830984 | -20.54774734 | 0.000164196 |
| *RESF1* | 2.83267605 | 4.149832769 | 20.51544289 | 0.000164196 |
| *LONP1* | -2.203651927 | 2.273418814 | -20.50836021 | 0.000164196 |
| *KIF1C* | -2.390783038 | 2.693460003 | -20.50525144 | 0.000164196 |
| *KIAA2013* | -2.843285555 | 3.307459104 | -20.4983355 | 0.000164196 |
| *NOC2L* | -2.179371386 | 2.920430794 | -20.4410218 | 0.000164196 |
| *DDRGK1* | -2.032847329 | 1.988948144 | -20.17617139 | 0.000166757 |
| *IL1RN* | -2.477025298 | 1.252659708 | -20.15640127 | 0.000166757 |
| *MZT2B* | -2.754568902 | 1.936036788 | -20.03648121 | 0.000166757 |
| *CD164* | 2.493326051 | 3.570850215 | 19.94641397 | 0.000166757 |
| *SLC66A2* | -2.099550128 | 2.044812065 | -19.91541161 | 0.000166757 |
| *ZGPAT* | -2.801346259 | 2.167864906 | -19.75153929 | 0.000166757 |
| *DRAP1* | -2.550656606 | 2.214617234 | -19.72435033 | 0.000166757 |
| *DAPK3* | -3.429596579 | 3.443988796 | -19.69348291 | 0.000166757 |
| *KRAS* | 2.031642424 | 2.41784848 | 19.67532216 | 0.000166757 |
| *RNH1* | -2.447886881 | 2.426820041 | -19.67400218 | 0.000166757 |
| *TNRC18* | -3.56266886 | 3.647510277 | -19.66861603 | 0.000166757 |
| *HES4* | -3.706360821 | 2.183250829 | -19.55985795 | 0.000168372 |
| *NES* | -3.365296652 | 4.121599182 | -19.53460284 | 0.000168607 |
| *COL4A2* | -2.592477816 | 4.291847431 | -19.52317131 | 0.000168607 |
| *TRA2A* | 2.064516872 | 3.758153919 | 19.4682333 | 0.000168607 |
| *ZNF428* | -3.510254007 | 2.68195643 | -19.44689458 | 0.000168946 |
| *THOP1* | -2.611340852 | 1.912705143 | -19.39344009 | 0.000169191 |
| *ARVCF* | -2.371915486 | 1.699357323 | -19.37074857 | 0.000169191 |
| *PRPF6* | -2.47767946 | 3.869120261 | -19.35204472 | 0.000169191 |
| *ARL6IP4* | -2.428345165 | 2.221103082 | -19.30476709 | 0.000169191 |
| *GABPA* | 2.265150653 | 1.799756504 | 19.28168243 | 0.000169191 |
| *CADM4* | -2.337805639 | 2.382777142 | -19.27975425 | 0.000169191 |
| *IRF7* | -2.359694237 | 1.870830738 | -19.2162856 | 0.000169531 |
| *HSDL2* | 2.093489833 | 2.31498176 | 19.16398707 | 0.000171435 |
| *MYH14* | -2.366772124 | 2.249034936 | -19.13383611 | 0.000172274 |
| *LMAN1* | 2.19784672 | 4.124790085 | 19.09168549 | 0.000173005 |
| *FAR1* | 2.229592689 | 2.247122867 | 19.08265843 | 0.000173005 |
| *TRAM1* | 2.489386692 | 4.309302487 | 19.02308006 | 0.000173005 |
| *BCAR1* | -2.200022322 | 1.937957409 | -18.93512557 | 0.000173005 |
| *REV3L* | 2.126773086 | 3.057784257 | 18.92867866 | 0.000173005 |
| *FAM122A* | 2.13703431 | 2.667763543 | 18.92688448 | 0.000173005 |
| *GIPC1* | -3.22590927 | 2.838981193 | -18.91341887 | 0.000173005 |
| *CCDC102A* | -2.751818059 | 2.261136147 | -18.87627895 | 0.000173005 |
| *IL6ST* | 2.788360181 | 3.902109759 | 18.87429085 | 0.000173005 |
| *AURKAIP1* | -3.087529486 | 2.097860634 | -18.84149062 | 0.000173005 |
| *RO60* | 2.003839478 | 2.621294074 | 18.81969535 | 0.000173005 |
| *AKAP17A* | -2.542357102 | 2.67572615 | -18.80342813 | 0.000173005 |
| *HOMER3* | -2.777298159 | 2.801594409 | -18.65022612 | 0.000173005 |
| *MEGF8* | -2.129966328 | 2.664373579 | -18.62185342 | 0.000173005 |
| *OGFR* | -2.790099633 | 2.247157097 | -18.56944925 | 0.000173005 |
| *ANTKMT* | -3.59944869 | 1.930960822 | -18.5639838 | 0.000173005 |
| *CC2D1A* | -2.087704174 | 2.301352442 | -18.53196743 | 0.000173005 |
| *TMEM123* | 2.506084834 | 3.798886823 | 18.48713893 | 0.000173166 |
| *SOX12* | -2.262509323 | 2.818070174 | -18.47342568 | 0.000173166 |
| *TSR3* | -3.050798007 | 2.311736025 | -18.34874104 | 0.00017537 |
| *POLRMT* | -2.420486615 | 2.551992612 | -18.34146793 | 0.00017537 |
| *MIIP* | -2.05431357 | 1.731198192 | -18.24784205 | 0.000177862 |
| *AHDC1* | -2.056515688 | 2.804990242 | -18.17065814 | 0.000178501 |
| *TOMM20* | 2.147561966 | 3.938922942 | 18.16470838 | 0.000178501 |
| *RBM42* | -2.370325749 | 2.588938598 | -18.08009829 | 0.000178501 |
| *FBXL19* | -2.538270084 | 2.492937033 | -17.97855252 | 0.000178617 |
| *MACROD1* | -2.188921891 | 1.805381213 | -17.96037957 | 0.000178617 |
| *SLC40A1* | 2.052059772 | 3.389351999 | 17.91014865 | 0.000180438 |
| *QSER1* | 2.135472737 | 3.127653047 | 17.87808954 | 0.000180997 |
| *CHMP6* | -2.035461573 | 2.58540805 | -17.84896678 | 0.000181195 |
| *SIKE1* | 2.09505615 | 2.49792326 | 17.81303806 | 0.000181448 |
| *P3H3* | -2.193889193 | 2.166300419 | -17.78661787 | 0.000182024 |
| *MOB1A* | 2.5579447 | 3.51236632 | 17.7675186 | 0.000182024 |
| *MCRIP1* | -2.471651883 | 1.79324996 | -17.69596868 | 0.000183486 |
| *SCAMP1* | 2.107893692 | 2.025429168 | 17.64187819 | 0.000185617 |
| *SDHAF1* | -2.365137457 | 2.786061403 | -17.62130099 | 0.000185848 |
| *SEMA3B* | -2.508174147 | 2.7867124 | -17.5502105 | 0.000187682 |
| *SRSF11* | 2.094962482 | 3.136065463 | 17.54857215 | 0.000187682 |
| *PLCB3* | -2.071680982 | 2.323142444 | -17.52506236 | 0.000187682 |
| *NRAS* | 2.217429448 | 2.976481581 | 17.49125216 | 0.000188217 |
| *TNKS1BP1* | -2.973496448 | 3.520003691 | -17.42768526 | 0.000189206 |
| *CCDC85B* | -5.068626859 | 3.364384617 | -17.41073308 | 0.000189206 |
| *AGRN* | -3.455497589 | 4.170613316 | -17.39250774 | 0.000189206 |
| *EEF1D* | -2.617545732 | 3.242622198 | -17.3881767 | 0.000189206 |
| *TMEM132A* | -2.53445916 | 2.678648804 | -17.33325668 | 0.000190196 |
| *GADD45GIP1* | -2.165948359 | 2.240524103 | -17.225247 | 0.000193094 |
| *SERINC1* | 2.414829337 | 3.78277862 | 17.13772282 | 0.000196019 |
| *ZNF865* | -2.056206525 | 1.63074612 | -17.13229497 | 0.000196019 |
| *SCAND1* | -3.140726926 | 2.222932077 | -17.09650522 | 0.000196143 |
| *ARL5B* | 2.165708579 | 2.198740533 | 17.03645605 | 0.000198526 |
| *CARD9* | -2.213466495 | 1.389239969 | -17.01186469 | 0.000198741 |
| *ARHGEF1* | -2.230257428 | 2.714020297 | -16.99195156 | 0.000198741 |
| *PON2* | 2.034566626 | 2.747682828 | 16.9908287 | 0.000198741 |
| *MZT2A* | -2.129550703 | 1.665790515 | -16.94871023 | 0.00020003 |
| *GNA11* | -2.146699136 | 2.416672265 | -16.93917886 | 0.00020003 |
| *ISOC2* | -2.017600488 | 1.665477919 | -16.92221169 | 0.00020003 |
| *PPDPF* | -2.590233247 | 2.422670602 | -16.91129333 | 0.000200293 |
| *RANGAP1* | -2.385748554 | 2.854977265 | -16.8566654 | 0.000200614 |
| *AP3S1* | 2.053389764 | 2.074735828 | 16.85278242 | 0.000200614 |
| *CPSF6* | 2.521513325 | 2.888254252 | 16.84056353 | 0.000200936 |
| *DDX17* | 2.048388181 | 5.952023053 | 16.78389261 | 0.000202242 |
| *HSPBP1* | -2.732404246 | 2.643414193 | -16.76773359 | 0.000202242 |
| *LMNB2* | -2.955674458 | 3.43367118 | -16.72635515 | 0.000202721 |
| *ETNK1* | 2.01727913 | 1.884341027 | 16.70838538 | 0.000202721 |
| *TP53I13* | -2.492810012 | 2.052150808 | -16.6487116 | 0.000202721 |
| *PPP1R16A* | -2.361671045 | 2.153606367 | -16.61857139 | 0.000202721 |
| *NAT14* | -3.265537835 | 2.280632343 | -16.61401428 | 0.000202721 |
| *UBXN6* | -2.012128539 | 2.226764426 | -16.56923285 | 0.000202721 |
| *NFKBIL1* | -2.569253243 | 2.0644611 | -16.50913139 | 0.000204478 |
| *ARHGEF40* | -2.523836732 | 3.251981612 | -16.48819836 | 0.000205086 |
| *EFNB1* | -2.459932492 | 2.231211291 | -16.48096584 | 0.000205173 |
| *INTS1* | -2.214311212 | 3.184597917 | -16.45452217 | 0.000205927 |
| *ECI1* | -2.865369541 | 2.88837409 | -16.43957121 | 0.000205982 |
| *DDIT4* | -2.039630938 | 2.239182068 | -16.4303002 | 0.000206215 |
| *MYH9* | -2.566544553 | 5.58204343 | -16.40852066 | 0.000206272 |
| *TPM2* | -2.785758437 | 2.631541317 | -16.35948254 | 0.000206714 |
| *LYPLA1* | 2.05379031 | 2.758860312 | 16.31392751 | 0.000208295 |
| *ELN* | -2.038655154 | 1.526536968 | -16.28986285 | 0.000209012 |
| *EPOP* | -2.850472597 | 1.850843786 | -16.28674878 | 0.000209012 |
| *PHRF1* | -2.508451014 | 3.025335603 | -16.2549496 | 0.000209146 |
| *SSR3* | 2.1148134 | 4.075733206 | 16.23989351 | 0.000209146 |
| *SH3GL1* | -2.730818108 | 3.148957028 | -16.21994987 | 0.000209146 |
| *ATP5F1C* | 2.225949141 | 2.739380797 | 16.1486297 | 0.000210323 |
| *NAPRT* | -2.399665744 | 2.15661697 | -16.13147962 | 0.000210724 |
| *BCL2A1* | -2.163856189 | 1.250555257 | -16.11532753 | 0.000210743 |
| *TECPR1* | -2.074107117 | 1.620972914 | -16.07847924 | 0.000211076 |
| *KRBA1* | -2.049199119 | 1.758806434 | -15.9836166 | 0.000214252 |
| *CCDC8* | -2.557067627 | 2.49229647 | -15.96746916 | 0.000214625 |
| *SSBP4* | -2.650054432 | 2.278619523 | -15.95096044 | 0.000215157 |
| *TRIM56* | -2.018663754 | 2.519673515 | -15.83341616 | 0.000220943 |
| *LAPTM4A* | 2.470214039 | 5.958345943 | 15.79981352 | 0.000221225 |
| *IL1B* | -2.469565705 | 1.514383314 | -15.7988714 | 0.000221225 |
| *SLC25A36* | 2.21697581 | 2.806619473 | 15.7869786 | 0.000221225 |
| *RNF187* | -2.410545485 | 3.144321439 | -15.77617299 | 0.000221225 |
| *ALAS2* | -2.240531927 | 1.325832174 | -15.77166844 | 0.000221225 |
| *SLC38A10* | -3.195598365 | 3.34837612 | -15.77108399 | 0.000221225 |
| *P3H4* | -2.037788287 | 2.507395661 | -15.69727959 | 0.000224735 |
| *ANKRD13B* | -2.367038489 | 2.769197528 | -15.67566726 | 0.00022601 |
| *PKN1* | -3.358201161 | 2.906950908 | -15.64023678 | 0.00022694 |
| *DPY19L1* | 3.048111912 | 3.300998002 | 15.62058564 | 0.000228124 |
| *RAB24* | -2.10915568 | 1.830218255 | -15.6158988 | 0.000228124 |
| *SIX5* | -2.908453309 | 2.517592608 | -15.54449437 | 0.000231616 |
| *COL18A1* | -3.588614141 | 3.952499985 | -15.52839178 | 0.000232549 |
| *ZDHHC21* | 2.155448551 | 1.912578826 | 15.46981756 | 0.000234585 |
| *GTPBP6* | -2.616781674 | 2.82946022 | -15.46706112 | 0.000234585 |
| *CEP250* | -2.004555939 | 1.850386063 | -15.41260108 | 0.000235868 |
| *COL16A1* | -2.514444196 | 3.061492404 | -15.41123344 | 0.000235868 |
| *IRF2BP1* | -3.133653677 | 3.023908492 | -15.39717855 | 0.000235932 |
| *PPP1R12C* | -3.313319858 | 2.996632525 | -15.38225176 | 0.000236547 |
| *GAS2L1* | -2.057046615 | 1.531572446 | -15.37227829 | 0.000236547 |
| *TMEM250* | -2.315245833 | 1.9523537 | -15.3287405 | 0.000238734 |
| *CCDC12* | -2.511303747 | 2.044235939 | -15.31596525 | 0.000239449 |
| *RABL6* | -2.14335268 | 2.275373113 | -15.30856738 | 0.000239709 |
| *KIF7* | -2.323015303 | 1.990059329 | -15.28064006 | 0.000240989 |
| *KIFC2* | -2.214490702 | 2.058967648 | -15.15450429 | 0.000247258 |
| *PTMS* | -3.157775893 | 5.147596287 | -15.13463414 | 0.000247258 |
| *MLLT1* | -2.101590868 | 2.514897305 | -15.1330121 | 0.000247258 |
| *SHARPIN* | -2.105273334 | 2.2185452 | -15.1285951 | 0.000247258 |
| *RECQL4* | -2.146460681 | 1.788459612 | -15.11711239 | 0.000247911 |
| *MEX3D* | -2.707675831 | 2.46338174 | -15.10729725 | 0.000248418 |
| *NPAT* | 2.127373779 | 1.91121388 | 15.06926372 | 0.000249624 |
| *C20orf27* | -2.415582522 | 2.541295222 | -15.01019516 | 0.000250911 |
| *FKBP8* | -2.954030707 | 3.617102702 | -14.99971568 | 0.000250911 |
| *TSNARE1* | -2.006936743 | 1.575287053 | -14.99253019 | 0.000250911 |
| *CCHCR1* | -2.315868162 | 2.090070514 | -14.98247472 | 0.000250911 |
| *TMEM106B* | 2.766663558 | 2.430777535 | 14.9748689 | 0.000250911 |
| *C21orf91* | 2.068589473 | 1.687050367 | 14.92687685 | 0.000252097 |
| *PAPOLA* | 2.509282515 | 3.529821731 | 14.89877754 | 0.000253616 |
| *CDR2L* | -2.741855754 | 2.558538244 | -14.89365085 | 0.000253724 |
| *TEDC1* | -2.141359026 | 1.390923247 | -14.88993104 | 0.000253724 |
| *CAVIN3* | -3.362268944 | 2.555363938 | -14.86258276 | 0.000255908 |
| *ATG101* | -2.627800437 | 2.126876788 | -14.80434523 | 0.000258164 |
| *CCNT2* | 2.166761721 | 2.106485521 | 14.80243224 | 0.000258164 |
| *NRIP1* | 2.108822531 | 2.595808834 | 14.74210689 | 0.000260773 |
| *METTL26* | -2.021542193 | 1.728948568 | -14.72302709 | 0.00026157 |
| *PALD1* | -2.107592425 | 1.869776115 | -14.7167558 | 0.00026157 |
| *TMED10* | 2.205616708 | 4.885172059 | 14.68721543 | 0.000262802 |
| *HSPA1B* | -2.349243723 | 4.228042079 | -14.66266837 | 0.000263988 |
| *DUS1L* | -2.129738435 | 2.633984468 | -14.61670836 | 0.000266735 |
| *PAK4* | -2.035291064 | 2.013029329 | -14.60452663 | 0.000267067 |
| *CTSO* | 2.630711805 | 3.09176849 | 14.54872236 | 0.000268727 |
| *PAXX* | -2.48687417 | 2.056210079 | -14.54564572 | 0.000268727 |
| *MAP1S* | -2.138495119 | 1.8972842 | -14.53952783 | 0.000268727 |
| *UBTF* | -2.350816773 | 3.088211513 | -14.45484027 | 0.000272815 |
| *RAB3IL1* | -2.490987296 | 2.968681818 | -14.44997097 | 0.000272964 |
| *TCEA2* | -2.039274714 | 2.133346071 | -14.43164393 | 0.00027345 |
| *RING1* | -2.009051095 | 3.073293862 | -14.40846306 | 0.00027465 |
| *AC119396* | -2.093443404 | 2.221868169 | -14.36975658 | 0.000276254 |
| *CDC34* | -2.398359407 | 1.98203598 | -14.31234147 | 0.000278049 |
| *LMNA* | -2.72510498 | 3.15705234 | -14.30742974 | 0.000278049 |
| *MRPS26* | -2.290469095 | 1.995586005 | -14.28035421 | 0.000278049 |
| *EIF3E* | 2.61642101 | 3.662995201 | 14.26888673 | 0.000278049 |
| *TYMP* | -2.393254858 | 2.199957881 | -14.2464918 | 0.000279067 |
| *ANKRD10* | 2.23734298 | 3.733063084 | 14.21958739 | 0.0002802 |
| *SAPCD2* | -2.640267452 | 1.856762494 | -14.20991813 | 0.0002802 |
| *ZNF22* | 2.307538116 | 2.858927355 | 14.18481292 | 0.000282284 |
| *ZNF579* | -2.526803224 | 1.722725001 | -14.16613713 | 0.000283964 |
| *LIMS1* | 2.036971737 | 3.448342959 | 14.10784422 | 0.000288307 |
| *LRFN4* | -2.605895625 | 1.876422329 | -14.07719938 | 0.000290405 |
| *PPP2R2D* | -2.05684028 | 2.540422971 | -14.07663274 | 0.000290405 |
| *TELO2* | -3.3640568 | 2.8488035 | -14.04497855 | 0.000290876 |
| *PWWP2B* | -2.773011691 | 2.200423782 | -13.9874299 | 0.000294434 |
| *ZNF770* | 2.426754511 | 3.217785694 | 13.96855331 | 0.000294434 |
| *LRP3* | -2.606435538 | 2.634412214 | -13.93275774 | 0.000297864 |
| *SSNA1* | -3.497219223 | 3.040965113 | -13.84882452 | 0.000303587 |
| *RHPN1* | -2.172907023 | 1.614451918 | -13.7618355 | 0.000309747 |
| *SPINDOC* | -2.143558712 | 2.169565837 | -13.74024494 | 0.00031116 |
| *MXRA8* | -2.791106554 | 3.684288511 | -13.7250109 | 0.000312338 |
| *CAPN15* | -2.671892062 | 2.320514871 | -13.69843571 | 0.000313545 |
| *CLK1* | 2.189710484 | 3.753147604 | 13.66856741 | 0.00031518 |
| *MIB2* | -2.660123957 | 2.547754671 | -13.65859673 | 0.00031518 |
| *VWA1* | -2.583946395 | 2.21354103 | -13.65267842 | 0.00031518 |
| *METRN* | -2.076214846 | 1.709607708 | -13.64727378 | 0.00031518 |
| *NOP53* | -3.364929541 | 3.752631407 | -13.57261693 | 0.000321099 |
| *PIGK* | 2.215964643 | 1.523660886 | 13.5520688 | 0.000321612 |
| *ASPSCR1* | -2.258936008 | 1.799065861 | -13.52895736 | 0.000323178 |
| *TNIP2* | -2.46182533 | 2.455911863 | -13.52338764 | 0.000323178 |
| *GPKOW* | -2.045161498 | 1.930360961 | -13.52173893 | 0.000323178 |
| *PBXIP1* | -2.754356607 | 3.38002035 | -13.48599598 | 0.000325907 |
| *VMA21* | 2.323609473 | 2.460991634 | 13.47675388 | 0.000326133 |
| *RAB18* | 2.231595148 | 2.289311126 | 13.44023767 | 0.000327331 |
| *CLK4* | 2.286550466 | 2.193391448 | 13.41839692 | 0.000329195 |
| *CAPZA1* | 2.579819989 | 3.887215747 | 13.39479778 | 0.000330292 |
| *AEBP1* | -2.023280098 | 3.059712047 | -13.34833905 | 0.000333691 |
| *HMGN4* | 2.230214205 | 3.524219287 | 13.32724721 | 0.00033598 |
| *STC1* | -2.277428838 | 2.247414678 | -13.29764899 | 0.000337811 |
| *EMILIN1* | -4.100398719 | 4.546312894 | -13.25825794 | 0.000339996 |
| *LSM4* | -2.206497461 | 2.102878419 | -13.2161868 | 0.000343131 |
| *TTYH3* | -2.567431872 | 3.486377013 | -13.20139584 | 0.000343846 |
| *SEPTIN5* | -2.126573685 | 1.749546773 | -13.14888354 | 0.000348904 |
| *EVA1B* | -3.184449071 | 1.676401921 | -13.12108348 | 0.000351133 |
| *MANEA* | 2.283292793 | 1.404949065 | 13.12072769 | 0.000351133 |
| *CEP131* | -2.814058469 | 2.13866712 | -13.11907308 | 0.000351133 |
| *CAMSAP3* | -2.016144867 | 1.527288634 | -13.11506369 | 0.000351241 |
| *GPSM1* | -2.7628025 | 2.908205231 | -13.09623014 | 0.000353414 |
| *MRPL41* | -2.263623281 | 3.449657461 | -13.07561786 | 0.000353773 |
| *RAMP1* | -2.33464214 | 2.63144083 | -12.94009409 | 0.000366327 |
| *CCS* | -2.070723361 | 2.377240128 | -12.92108283 | 0.000368654 |
| *MFSD3* | -2.281794559 | 1.985066045 | -12.90676107 | 0.000369389 |
| *TPGS1* | -2.146589921 | 1.461323671 | -12.88454741 | 0.000370716 |
| *TIMM29* | -2.041466923 | 1.653766871 | -12.83436762 | 0.000373145 |
| *TRIM11* | -2.213102695 | 2.268777046 | -12.83350883 | 0.000373145 |
| *LUM* | 3.58844565 | 6.17766248 | 12.80566276 | 0.000375806 |
| *CITED4* | -3.416620202 | 3.096944364 | -12.77627514 | 0.000378748 |
| *CCNH* | 2.227467177 | 2.139520689 | 12.7468257 | 0.00038105 |
| *ZBTB7B* | -2.005350671 | 2.118912775 | -12.72684338 | 0.000382736 |
| *GMFB* | 2.096179072 | 2.043484861 | 12.72655812 | 0.000382736 |
| *CNPY3* | -2.124374227 | 2.310193138 | -12.7107249 | 0.000383108 |
| *C1QTNF5* | -2.785559731 | 1.835469276 | -12.70851352 | 0.000383108 |
| *HSPA12B* | -2.28089262 | 2.243915528 | -12.68153764 | 0.000383847 |
| *GPC1* | -2.156388373 | 2.893945833 | -12.67873284 | 0.000383847 |
| *TTC14* | 2.076451181 | 2.326633161 | 12.64503122 | 0.000386559 |
| *GADD45G* | -2.629030796 | 1.822755768 | -12.63079947 | 0.000387286 |
| *TMEM126A* | 2.267311277 | 1.887984366 | 12.62516624 | 0.000387537 |
| *RCC1L* | -2.03271857 | 2.17651664 | -12.61917405 | 0.000387567 |
| *AKAP11* | 2.22801156 | 3.224040336 | 12.61900727 | 0.000387567 |
| *RBMX* | 2.434217852 | 4.221335347 | 12.61751877 | 0.000387567 |
| *PRXL2B* | -2.111202989 | 2.136252851 | -12.59657791 | 0.000388605 |
| *CPSF1* | -2.051447527 | 3.128826832 | -12.59137639 | 0.000388742 |
| *LMF2* | -2.199276025 | 3.015049158 | -12.56595056 | 0.000389929 |
| *IRF3* | -2.325671456 | 2.69072714 | -12.56096521 | 0.000389929 |
| *SBF1* | -2.322890203 | 2.891138444 | -12.51935619 | 0.000393044 |
| *ABHD17A* | -2.765053172 | 2.595982941 | -12.4949053 | 0.000395907 |
| *CAPS* | -3.239129841 | 2.848033159 | -12.47794187 | 0.000396822 |
| *CDC37* | -2.868782055 | 2.876313873 | -12.47783484 | 0.000396822 |
| *PRPF39* | 2.022359397 | 1.750431404 | 12.45352357 | 0.000397983 |
| *R3HCC1* | -2.260007409 | 2.165911284 | -12.40896217 | 0.000402366 |
| *IKBKG* | -2.04192803 | 1.672075326 | -12.39189415 | 0.000403152 |
| *DBN1* | -2.244280438 | 3.329664217 | -12.39112119 | 0.000403152 |
| *CD248* | -2.469628966 | 5.64220577 | -12.36769481 | 0.000404607 |
| *CCDC14* | 2.330820306 | 2.887647745 | 12.33987931 | 0.000406869 |
| *FAM207A* | -2.301793213 | 1.490108815 | -12.22728667 | 0.000419224 |
| *IRAK1* | -2.030608937 | 2.672646659 | -12.20722257 | 0.000420924 |
| *RHBDD3* | -2.087726452 | 2.144883264 | -12.18676406 | 0.000423308 |
| *FJX1* | -2.617216863 | 4.200156897 | -12.18289907 | 0.000423308 |
| *ILVBL* | -2.086293359 | 1.857517438 | -12.18190747 | 0.000423308 |
| *KMO* | 2.379138054 | 1.844338721 | 12.14733479 | 0.000428497 |
| *DPY19L4* | 2.327477841 | 1.899767905 | 12.13606762 | 0.000429116 |
| *LZTS2* | -2.502149327 | 2.610830981 | -12.1266189 | 0.000429116 |
| *NOTCH3* | -2.274742115 | 3.138740041 | -12.12491763 | 0.000429116 |
| *H2AX* | -2.471208098 | 3.492368491 | -12.09824385 | 0.000432525 |
| *RPL15* | 2.291475068 | 4.710027147 | 12.02697363 | 0.000440156 |
| *TSEN34* | -2.014643864 | 2.034355736 | -12.02364844 | 0.000440156 |
| *EMC10* | -2.384655454 | 2.443044449 | -11.97712622 | 0.000446681 |
| *PEX6* | -2.355417717 | 2.641427827 | -11.95907233 | 0.000448581 |
| *RABEP2* | -2.846411666 | 2.085502457 | -11.95779691 | 0.000448581 |
| *ARMC5* | -2.213655691 | 1.695833095 | -11.94966193 | 0.00044914 |
| *EXOSC5* | -2.294933921 | 2.001174622 | -11.94129256 | 0.000449903 |
| *PNMA6A* | -2.401614882 | 1.355904894 | -11.93096924 | 0.000450884 |
| *ATF6B* | -2.976092575 | 3.471273212 | -11.89834572 | 0.000454137 |
| *CRELD2* | -2.272723829 | 2.076193821 | -11.89057798 | 0.00045539 |
| *TMED1* | -2.45210446 | 2.179489527 | -11.86721564 | 0.000458847 |
| *RARRES2* | -2.523867468 | 2.989038001 | -11.86097622 | 0.000459358 |
| *NHLRC2* | 2.007925626 | 2.58887943 | 11.85543501 | 0.000459358 |
| *CYBA* | -2.432889779 | 2.380319888 | -11.8511919 | 0.00045945 |
| *PKD1* | -2.175276446 | 2.791129439 | -11.83382026 | 0.000461032 |
| *BORCS6* | -2.432934014 | 1.671864954 | -11.82482325 | 0.00046239 |
| *NUDT21* | 2.057268257 | 3.337331743 | 11.7970518 | 0.000464801 |
| *LTBP3* | -2.182176336 | 3.308333832 | -11.79360747 | 0.000464801 |
| *PPP1R14B* | -2.000084979 | 1.643959599 | -11.79355122 | 0.000464801 |
| *SLC4A2* | -2.108458601 | 2.682385523 | -11.76069069 | 0.000467687 |
| *ZNF787* | -2.193673066 | 1.818286871 | -11.76054336 | 0.000467687 |
| *CRABP2* | -2.013189244 | 4.481501636 | -11.7503364 | 0.000469527 |
| *DNAJC10* | 2.261923716 | 3.165214488 | 11.70153836 | 0.000476208 |
| *VPS13C* | 2.321039312 | 3.173657043 | 11.70053026 | 0.000476208 |
| *B4GALNT4* | -3.597278644 | 3.444503402 | -11.66766253 | 0.000480001 |
| *TRABD* | -2.438721639 | 2.221716672 | -11.63812342 | 0.000483357 |
| *RPL29* | 2.157147886 | 5.074135772 | 11.62016961 | 0.000484745 |
| *CRIP2* | -2.337624609 | 2.349357203 | -11.61745514 | 0.000485011 |
| *LAMTOR4* | -2.499821032 | 1.940137436 | -11.61135724 | 0.000485016 |
| *HIC1* | -2.514127279 | 2.672988143 | -11.60940982 | 0.000485113 |
| *ESRRA* | -2.309460577 | 2.009365121 | -11.58688238 | 0.000487891 |
| *MYBBP1A* | -2.166444313 | 2.884455317 | -11.57728354 | 0.000488652 |
| *NPDC1* | -2.843564028 | 2.844765707 | -11.54169941 | 0.000492281 |
| *PMS1* | 2.1688222 | 1.942170902 | 11.52233804 | 0.000494345 |
| *RMDN3* | -2.164668426 | 2.020570185 | -11.51336322 | 0.000495393 |
| *SELENOP* | 2.560479799 | 3.351277369 | 11.51051568 | 0.00049571 |
| *LRRC40* | 2.176523622 | 1.941526321 | 11.50125149 | 0.00049727 |
| *LRRC47* | -2.412863129 | 2.663400872 | -11.47884205 | 0.000500339 |
| *THEM6* | -2.432658049 | 2.302276218 | -11.3953318 | 0.000509542 |
| *PKP3* | -2.259831775 | 1.88720778 | -11.35343038 | 0.000516583 |
| *NOC4L* | -2.093558288 | 1.770722858 | -11.34500212 | 0.000517852 |
| *YDJC* | -2.28061819 | 2.162112756 | -11.30156114 | 0.000523158 |
| *TMEM259* | -2.364699932 | 3.616438265 | -11.27578801 | 0.000526825 |
| *ZNF644* | 2.130330884 | 2.684292613 | 11.26283612 | 0.000529336 |
| *BX255925* | -2.596237073 | 1.892460968 | -11.24229648 | 0.000531123 |
| *SOCS4* | 2.249102531 | 1.815998036 | 11.22127994 | 0.000534047 |
| *PTP4A1* | 2.557765512 | 3.728663607 | 11.20215063 | 0.000537577 |
| *NAGPA* | -2.044875177 | 1.484488646 | -11.19128838 | 0.000539654 |
| *SAMD11* | -3.176911494 | 2.66333028 | -11.16750446 | 0.000544186 |
| *FOXJ1* | -2.188158008 | 2.257528429 | -11.15710995 | 0.000545251 |
| *ZNF467* | -2.046481905 | 2.021447388 | -11.11675465 | 0.000553235 |
| *SLC52A2* | -2.14606028 | 1.710350244 | -11.09241107 | 0.000557628 |
| *FAM50A* | -2.109724229 | 1.749006751 | -11.08984444 | 0.000557965 |
| *COL5A3* | -2.646134448 | 1.910576487 | -11.06798792 | 0.000561746 |
| *MDK* | -2.278048918 | 4.392297063 | -11.05289749 | 0.000564786 |
| *SELENOF* | 2.288419798 | 4.722246496 | 11.00886112 | 0.000572281 |
| *MPND* | -2.373447935 | 1.833696526 | -10.89993912 | 0.000589989 |
| *ARFGAP1* | -2.0609559 | 2.91404181 | -10.898807 | 0.000589989 |
| *CPM* | 2.213306159 | 4.101432291 | 10.89245436 | 0.000590606 |
| *COL7A1* | -3.508014951 | 5.176718063 | -10.84359489 | 0.000597818 |
| *C8orf82* | -2.03906141 | 1.743867443 | -10.81466534 | 0.000602283 |
| *SBNO2* | -2.087066475 | 2.264263102 | -10.79489124 | 0.000604946 |
| *ZNF622* | -2.188214847 | 2.395231089 | -10.76592285 | 0.000610055 |
| *CUEDC2* | -2.140496547 | 2.931442119 | -10.7477123 | 0.000613546 |
| *RAB11FIP3* | -2.086199158 | 2.298986011 | -10.73621163 | 0.000614758 |
| *PHLDA3* | -2.026729847 | 1.879244781 | -10.72280815 | 0.000617579 |
| *CENPB* | -3.104696562 | 3.283348208 | -10.70821834 | 0.000619741 |
| *KMT5C* | -2.055171778 | 1.47983982 | -10.70150551 | 0.000620732 |
| *NR2F6* | -2.186822846 | 1.951088664 | -10.69249989 | 0.00062136 |
| *PPP1R35* | -3.690091588 | 2.578788284 | -10.67059878 | 0.000624212 |
| *TRIOBP* | -2.060285333 | 2.556436727 | -10.66261699 | 0.000625267 |
| *JOSD2* | -2.302262238 | 1.340242306 | -10.65102724 | 0.000626091 |
| *SLX1A* | -2.56908193 | 2.03462337 | -10.59402872 | 0.000636485 |
| *TLNRD1* | -2.564397239 | 2.338161141 | -10.57978767 | 0.000638872 |
| *CACFD1* | -2.230678269 | 1.77646428 | -10.52704105 | 0.000646241 |
| *STUB1* | -2.221920837 | 2.802750964 | -10.51757406 | 0.000646585 |
| *PIAS4* | -2.045393597 | 1.917534476 | -10.4999258 | 0.000650239 |
| *ARFRP1* | -2.212936795 | 1.925455629 | -10.46566881 | 0.000655989 |
| *SCRN2* | -2.541986251 | 2.287476243 | -10.43020826 | 0.000663997 |
| *PRR12* | -2.262717052 | 2.22895814 | -10.41515247 | 0.000666773 |
| *SPON2* | -2.83820405 | 3.258901105 | -10.39927322 | 0.000670389 |
| *TNFAIP2* | -2.062913824 | 2.878943448 | -10.36305143 | 0.000678327 |
| *EPS8L1* | -2.368395959 | 1.933904368 | -10.35841097 | 0.000679071 |
| *TMUB1* | -2.613540421 | 2.0055523 | -10.34519703 | 0.000681868 |
| *PRKCSH* | -2.057121531 | 3.838677401 | -10.31721088 | 0.000689372 |
| *AAMP* | -2.133039888 | 2.664423773 | -10.27767395 | 0.000698549 |
| *EGFL7* | -2.855417788 | 2.179400692 | -10.2719939 | 0.000699479 |
| *MVD* | -2.298331224 | 1.516960902 | -10.26065003 | 0.00070212 |
| *PSMA3* | 2.004167659 | 2.73340923 | 10.2551397 | 0.000703018 |
| *CENPX* | -2.604904354 | 2.455410989 | -10.21689449 | 0.000709179 |
| *PACSIN3* | -2.174037222 | 1.984712053 | -10.1896945 | 0.000715307 |
| *SPTSSA* | 2.172866031 | 1.912605726 | 10.15246428 | 0.000725206 |
| *NDUFA1* | -2.120724973 | 2.130103713 | -10.13185398 | 0.000730065 |
| *SSC5D* | -2.141484389 | 1.648419305 | -10.12634881 | 0.000731052 |
| *FBLN2* | -2.014725176 | 2.576664334 | -10.0781095 | 0.000741842 |
| *ZNF444* | -2.111260037 | 1.738772614 | -10.06287403 | 0.000745554 |
| *CLDN3* | -3.331172403 | 3.559621591 | -10.05978388 | 0.000745679 |
| *CCNI* | 2.096034172 | 5.076015628 | 10.05874457 | 0.000745679 |
| *UBXN1* | -2.049642366 | 2.975362378 | -10.02666759 | 0.000754115 |
| *MAP7D1* | -2.474398162 | 3.037308234 | -10.02663862 | 0.000754115 |
| *PELP1* | -2.053317264 | 2.534301504 | -10.01293443 | 0.000757457 |
| *CLIC4* | 2.009052041 | 4.477960325 | 9.998070614 | 0.000761162 |
| *SGCB* | 2.061640524 | 2.353587938 | 9.98385572 | 0.000763888 |
| *PES1* | -2.004323489 | 3.182078353 | -9.976437028 | 0.000765135 |
| *C5orf51* | 2.358823514 | 2.369401502 | 9.937522798 | 0.000775452 |
| *NUDT16L1* | -2.162293749 | 2.055689656 | -9.884858623 | 0.000785073 |
| *FBL* | -2.627147549 | 4.059177285 | -9.854551536 | 0.000791066 |
| *CD24* | 2.661011526 | 5.871481088 | 9.849609106 | 0.000792786 |
| *LIN7C* | 2.192942531 | 2.642903029 | 9.81418156 | 0.000801145 |
| *LZTS3* | -2.339279045 | 2.556725053 | -9.783893981 | 0.000809353 |
| *OBSL1* | -2.201933021 | 3.180023972 | -9.779291929 | 0.000810181 |
| *PHETA2* | -2.399094261 | 2.049167424 | -9.742791868 | 0.000821111 |
| *BRAT1* | -2.558127578 | 2.443202485 | -9.731811311 | 0.000824213 |
| *LAMA5* | -2.206195841 | 3.201985454 | -9.6989295 | 0.000831845 |
| *C5orf15* | 2.738036714 | 3.148198869 | 9.682633829 | 0.000836171 |
| *ITM2B* | 2.030911049 | 4.23475597 | 9.679258418 | 0.000836558 |
| *CCNG1* | 2.287162653 | 3.288125747 | 9.674743926 | 0.000836717 |
| *SESN3* | 2.947009493 | 3.925842778 | 9.666962385 | 0.000838786 |
| *CARD10* | -2.131542655 | 1.826782473 | -9.652234807 | 0.000841993 |
| *CRACR2B* | -2.045773374 | 1.733213874 | -9.640971049 | 0.000843013 |
| *FBXL15* | -2.853490225 | 2.096633504 | -9.607536912 | 0.000851878 |
| *ISG15* | -2.645688379 | 2.470501896 | -9.572437324 | 0.000862039 |
| *EXD3* | -2.060589757 | 1.701290126 | -9.48617305 | 0.00088867 |
| *TSSC4* | -2.054139715 | 1.765795474 | -9.464998625 | 0.000896636 |
| *ENGASE* | -2.124170313 | 2.172502134 | -9.463537807 | 0.000896768 |
| *LRRC45* | -2.001375592 | 1.596115336 | -9.444787905 | 0.000903232 |
| *ENKD1* | -2.56758107 | 1.796331216 | -9.415440948 | 0.000913943 |
| *TRMT61A* | -2.024825085 | 1.904908758 | -9.364751532 | 0.000932778 |
| *CLTA* | -2.241096941 | 3.540609925 | -9.318515675 | 0.000947922 |
| *CGGBP1* | 2.192409503 | 3.195479157 | 9.295956224 | 0.000955045 |
| *CTU1* | -2.473622839 | 1.641461845 | -9.285636009 | 0.000958343 |
| *ENDOG* | -3.430275748 | 2.294192279 | -9.237956355 | 0.000974734 |
| *RHOD* | -2.308480948 | 1.885766021 | -9.229969812 | 0.000978527 |
| *RPS6KA4* | -2.349513105 | 2.123036663 | -9.212911525 | 0.000983308 |
| *ALYREF* | -2.146526593 | 2.237564626 | -9.196843607 | 0.000988557 |
| *RHOT2* | -2.266514466 | 3.079673009 | -9.18355543 | 0.000993705 |
| *CD2BP2* | -2.011012092 | 2.905782775 | -9.1506034 | 0.001003401 |
| *ADRA2C* | -3.327067888 | 3.438484815 | -9.138673063 | 0.0010074 |
| *HLA-F* | -2.158059946 | 3.10366001 | -9.129561667 | 0.001008975 |
| *KLF16* | -2.284598139 | 1.616272537 | -9.124501503 | 0.001010737 |
| *ARHGDIA* | -2.202621208 | 3.624484213 | -9.120555922 | 0.001011739 |
| *HES6* | -2.619762855 | 1.316199307 | -9.117240909 | 0.001012436 |
| *SAC3D1* | -2.361364516 | 1.289235698 | -9.041640912 | 0.001039873 |
| *NDUFB7* | -2.338723575 | 3.142215317 | -8.93421903 | 0.001079872 |
| *TMEM129* | -2.166242675 | 2.243088528 | -8.928204582 | 0.001082839 |
| *NPW* | -2.737365527 | 2.081043577 | -8.924801694 | 0.001083737 |
| *TSEN54* | -2.20595438 | 2.038399566 | -8.9107959 | 0.00108989 |
| *ORMDL1* | 2.590110754 | 2.426754802 | 8.886700515 | 0.001099712 |
| *MRPS34* | -2.63812268 | 3.456811161 | -8.861438039 | 0.001109358 |
| *SYPL1* | 2.163536386 | 3.483906852 | 8.77672543 | 0.001142375 |
| *FSCN1* | -2.034588542 | 4.017338838 | -8.763416164 | 0.001147468 |
| *SPINT2* | -2.373880858 | 3.21522992 | -8.741154907 | 0.00115945 |
| *SNX3* | 2.663952372 | 3.998514534 | 8.738810706 | 0.001159778 |
| *ARF4* | 2.236857871 | 4.751946635 | 8.706816013 | 0.001172579 |
| *REEP3* | 2.241470971 | 2.839100201 | 8.673352535 | 0.001188895 |
| *TMEM200B* | -2.165017041 | 1.112862466 | -8.655499 | 0.001194882 |
| *TMEM230* | 2.28968695 | 2.520210328 | 8.637834315 | 0.001203134 |
| *CLTB* | -2.157216196 | 2.038505213 | -8.608021764 | 0.001213099 |
| *BOK* | -2.79787948 | 2.554850785 | -8.606809274 | 0.001213099 |
| *RWDD4* | 2.154009917 | 1.815005113 | 8.564721807 | 0.001232488 |
| *CDKN2C* | -2.293384807 | 2.534167083 | -8.545770947 | 0.001240458 |
| *NAB2* | -2.290659357 | 2.960787509 | -8.535138271 | 0.001245124 |
| *CDC42EP5* | -2.283580232 | 1.532631968 | -8.529573676 | 0.001248472 |
| *SHOC2* | 2.144576716 | 2.05700693 | 8.525823786 | 0.001250007 |
| *COL1A1* | -2.087597577 | 9.13333854 | -8.497593306 | 0.001265502 |
| *PHETA1* | -2.395299075 | 1.936874704 | -8.451269538 | 0.001295597 |
| *CHPF* | -2.320368569 | 3.337305587 | -8.438786857 | 0.001303307 |
| *NT5C* | -2.03654488 | 1.980458872 | -8.382553767 | 0.001331246 |
| *RAB14* | 2.129960342 | 3.64735622 | 8.355591593 | 0.001347199 |
| *LAMTOR3* | 2.042756368 | 2.199498805 | 8.321797522 | 0.00136646 |
| *TWIST1* | -2.090737882 | 1.836520316 | -8.313496738 | 0.001371218 |
| *PCNP* | 2.35348399 | 3.38115295 | 8.296687438 | 0.001379129 |
| *PNRC2* | 2.022389747 | 4.067837091 | 8.289506691 | 0.00138322 |
| *TRADD* | -2.759105497 | 1.945409035 | -8.232820582 | 0.001417266 |
| *PRPF31* | -2.024609111 | 2.789959541 | -8.215587385 | 0.001426419 |
| *MRPL23* | -2.756824959 | 2.278224712 | -8.203500354 | 0.001433645 |
| *DOK7* | -2.13756372 | 2.025254428 | -8.171907024 | 0.001454317 |
| *DEDD2* | -2.019340761 | 2.001364688 | -8.171231096 | 0.001454317 |
| *HSPB1* | -2.005570062 | 3.802354687 | -8.129085539 | 0.001478354 |
| *TWF1* | 2.359647349 | 3.050164298 | 8.116271409 | 0.001487066 |
| *PPP1R14A* | -2.390740122 | 2.189038257 | -8.091878079 | 0.00150256 |
| *PIM3* | -2.555844082 | 2.571018251 | -8.072844567 | 0.001517022 |
| *CROCC2* | -2.079473341 | 1.261829635 | -8.062348703 | 0.001522339 |
| *NR1D2* | 2.03885489 | 2.830806331 | 8.007743479 | 0.001564603 |
| *TWSG1* | 2.042038592 | 4.016961207 | 7.944436269 | 0.00161094 |
| *SHROOM1* | -2.079557618 | 2.343813542 | -7.892780015 | 0.001653986 |
| *CMTM6* | 2.105918237 | 4.696523356 | 7.889657226 | 0.001656322 |
| *FOXL2* | -2.455931552 | 4.194406345 | -7.872333984 | 0.001667864 |
| *H1-10* | -2.631626089 | 2.645548014 | -7.840484606 | 0.001694043 |
| *ARHGAP45* | -2.07576066 | 1.539691706 | -7.784636538 | 0.001738067 |
| *FAM83E* | -2.06005729 | 1.232292051 | -7.7708169 | 0.001750249 |
| *FAM83H* | -2.039211813 | 1.922379974 | -7.76061179 | 0.001759949 |
| *IGFBP2* | -2.007010912 | 3.266494877 | -7.746651794 | 0.001770349 |
| *ZNF219* | -2.077034905 | 1.717555132 | -7.739762192 | 0.001776832 |
| *KANK3* | -2.367600554 | 1.394564728 | -7.709629635 | 0.001805621 |
| *RASSF7* | -2.183305239 | 1.729199251 | -7.672689744 | 0.001836192 |
| *ALKBH7* | -2.19605462 | 2.117627946 | -7.606775395 | 0.00189679 |
| *TMEM167A* | 2.331585581 | 3.044141615 | 7.557146991 | 0.001940251 |
| *H2BC4* | 2.723609797 | 4.152774367 | 7.545259415 | 0.001949571 |
| *ZNF680* | 2.065096171 | 1.767239031 | 7.541776029 | 0.001951392 |
| *CCNL1* | 2.121851799 | 2.445628416 | 7.418775884 | 0.002065571 |
| *C1orf122* | -2.749528455 | 1.975200657 | -7.409066538 | 0.002075278 |
| *HSPB6* | -2.289884122 | 2.086209406 | -7.40533826 | 0.00207943 |
| *EXOC3L2* | -2.054827686 | 1.543487606 | -7.394029994 | 0.002089173 |
| *ZBTB12* | -2.269422497 | 2.260251338 | -7.345075481 | 0.002141099 |
| *DYNLT3* | 2.388994674 | 1.911002653 | 7.325468443 | 0.002162121 |
| *ROMO1* | -2.216193992 | 2.072776946 | -7.319734171 | 0.002167558 |
| *EMID1* | -2.303341004 | 2.213656943 | -7.314698755 | 0.00217395 |
| *CLEC11A* | -3.021947151 | 3.441054485 | -7.266944826 | 0.002227109 |
| *STX10* | -2.043243895 | 2.65918391 | -7.234968545 | 0.00225455 |
| *TK1* | -2.445502533 | 2.725205457 | -7.196423717 | 0.002301308 |
| *TPPP3* | -2.008114925 | 2.661137221 | -7.171718315 | 0.002331812 |
| *IGSF8* | -2.175107605 | 2.929752572 | -7.075337876 | 0.002445685 |
| *HSD17B14* | -2.082707958 | 1.677968709 | -6.973611724 | 0.002578115 |
| *LTBP4* | -2.552769898 | 5.14461844 | -6.966913748 | 0.002587341 |
| *DUSP23* | -2.170643042 | 1.91123577 | -6.911006386 | 0.002657309 |
| *SNRNP70* | -2.106442342 | 3.793798884 | -6.810967773 | 0.002803239 |
| *CSE1L* | 2.169150602 | 3.579060379 | 6.805447848 | 0.00280976 |
| *TMSB10* | -2.053050822 | 5.280372721 | -6.794128939 | 0.002827704 |
| *ZSWIM9* | -2.069970174 | 1.434094293 | -6.787301188 | 0.002839565 |
| *PCSK1N* | -2.645772907 | 1.446805859 | -6.69398868 | 0.002985332 |
| *PFDN2* | -2.019316315 | 1.536532381 | -6.677516785 | 0.003007716 |
| *IHH* | -2.040831753 | 2.750107786 | -6.614682035 | 0.003115518 |
| *COL9A2* | -2.567714101 | 2.127738103 | -6.583128488 | 0.003174403 |
| *PGGHG* | -2.055622691 | 2.353929506 | -6.502955266 | 0.003319308 |
| *HPS6* | -2.361801663 | 2.075256391 | -6.492356743 | 0.003336968 |
| *LAMC3* | -2.608377198 | 3.305432274 | -6.483332947 | 0.003351002 |
| *PGRMC1* | 2.005343604 | 7.002423538 | 6.406878773 | 0.003497083 |
| *BEX4* | 2.751548938 | 3.19167931 | 6.332439631 | 0.003649557 |
| *NME3* | -2.275314707 | 2.235422629 | -6.247413193 | 0.003848802 |
| *H3C7* | -2.164999462 | 2.902969485 | -6.246811047 | 0.003849775 |
| *DTX1* | -2.02337569 | 2.607388551 | -6.073785308 | 0.00429621 |
| *DEGS1* | 2.263479362 | 2.560610549 | 6.021755159 | 0.004433782 |
| *H1-4* | -2.035220463 | 4.612052694 | -5.951227889 | 0.00461511 |
| *TMEM69* | 2.039425615 | 2.334085238 | 5.945421546 | 0.004632289 |
| *CSRNP1* | -2.125467256 | 2.222344382 | -5.92247641 | 0.00469585 |
| *KRT7* | -2.046583084 | 1.969957623 | -5.787384221 | 0.005132504 |
| *ANAPC4* | 2.124750324 | 3.545002494 | 5.730390771 | 0.005335633 |
| *TIPRL* | 2.066055211 | 2.900902583 | 5.716002869 | 0.005386759 |
| *TFPT* | -2.095115878 | 1.714506359 | -5.526633732 | 0.006085131 |
| *RAB8B* | 2.106978258 | 2.409409771 | 5.375442157 | 0.006767511 |
| *EDNRA* | 2.014660742 | 3.458702605 | 5.375276783 | 0.006767511 |
| *C19orf71* | -2.497679659 | 1.718083034 | -5.230338387 | 0.00747264 |
| *MSLN* | -2.193833514 | 1.862099615 | -5.029359576 | 0.008623946 |
| *TMEM160* | -3.26054402 | 1.696441707 | -4.976262984 | 0.008979473 |
| *NUDT18* | -2.079051355 | 1.554009264 | -4.912334384 | 0.009398409 |
| *GLIPR1* | 2.286967066 | 3.219724601 | 4.895372591 | 0.009519808 |
| *C2CD4B* | -2.215270352 | 1.380106157 | -4.814749119 | 0.010132842 |
| *SDF2L1* | -2.107172525 | 1.417763141 | -4.570777998 | 0.012183333 |
| *SLC9A3R1* | -2.020970831 | 4.429463192 | -4.535478976 | 0.012523386 |
| *SOD3* | -2.110125523 | 1.462542552 | -4.523039845 | 0.012643275 |
| *ACTA1* | -2.013583104 | 1.025623226 | -4.452092206 | 0.013377179 |
| *TRH* | -2.156857374 | 2.996212199 | -4.271716853 | 0.01558397 |
| *EHF* | 2.124392211 | 3.518818854 | 4.178478064 | 0.016838506 |
| *AP001453* | -2.066683284 | 1.682347062 | -4.070444885 | 0.018531731 |
| *HPGD* | 2.39854831 | 1.530331471 | 3.771228054 | 0.024328725 |
| *LAGE3* | -2.035010134 | 1.325835284 | -3.6574404 | 0.027013067 |
| *ZNF593* | -2.03647418 | 2.170576481 | -3.625265059 | 0.027784019 |
| *SFTPA2* | -2.43015842 | 1.21507921 | -3.219312949 | 0.041318055 |
| *IER2* | -2.215171728 | 3.06627565 | -3.110972447 | 0.045985474 |
| *SFTPA1* | -2.164346142 | 1.107085003 | -3.063896588 | 0.048320987 |

The filtering criteria of p-value < 0.05 and | log2 fold change (FC)| > 2.

**Supplementary Table 4. The results of Gene Ontology (GO) term enrichment analysis.**

| **Type** | **ID** | **Description** | **GeneRatio** | **BgRatio** | **pvalue** | **qvalue** | **Count** |
| --- | --- | --- | --- | --- | --- | --- | --- |
| BP | GO:0030198 | extracellular matrix organization | 24/580 | 318/18903 | 5.06E-05 | 0.06202428 | 24 |
| BP | GO:0043062 | extracellular structure organization | 24/580 | 319/18903 | 5.32E-05 | 0.06202428 | 24 |
| BP | GO:0008380 | RNA splicing | 31/580 | 469/18903 | 5.60E-05 | x | 31 |
| BP | GO:0045229 | external encapsulating structure organization | 24/580 | 321/18903 | 5.87E-05 | 0.06202428 | 24 |
| BP | GO:0051647 | nucleus localization | 6/580 | 28/18903 | 0.00017214 | 0.14549965 | 6 |
| BP | GO:0035088 | establishment or maintenance of apical/basal cell polarity | 8/580 | 55/18903 | 0.00025567 | 0.15436016 | 8 |
| BP | GO:0061245 | establishment or maintenance of bipolar cell polarity | 8/580 | 55/18903 | 0.00025567 | 0.15436016 | 8 |
| BP | GO:0044380 | protein localization to cytoskeleton | 8/580 | 58/18903 | 0.00037137 | 0.19618836 | 8 |
| CC | GO:0005581 | collagen trimer | 14/604 | 86/19869 | 3.00E-07 | 0.00015291 | 14 |
| CC | GO:0098644 | complex of collagen trimers | 7/604 | 22/19869 | 2.65E-06 | 0.00067767 | 7 |
| CC | GO:0005583 | fibrillar collagen trimer | 5/604 | 12/19869 | 1.69E-05 | 0.00216169 | 5 |
| CC | GO:0098643 | banded collagen fibril | 5/604 | 12/19869 | 1.69E-05 | 0.00216169 | 5 |
| CC | GO:0062023 | collagen-containing extracellular matrix | 28/604 | 433/19869 | 0.00015847 | 0.01618055 | 28 |
| CC | GO:0030687 | preribosome, large subunit precursor | 5/604 | 21/19869 | 0.00034671 | 0.02950085 | 5 |
| CC | GO:0005788 | endoplasmic reticulum lumen | 21/604 | 312/19869 | 0.00060798 | 0.04434171 | 21 |
| CC | GO:0030135 | coated vesicle | 20/604 | 310/19869 | 0.00135946 | 0.08675522 | 20 |
| CC | GO:0030686 | 90S preribosome | 5/604 | 29/19869 | 0.0016555 | 0.09390868 | 5 |
| CC | GO:0030684 | preribosome | 8/604 | 76/19869 | 0.00213484 | 0.09889539 | 8 |
| CC | GO:0097525 | spliceosomal snRNP complex | 9/604 | 95/19869 | 0.00240779 | 0.09889539 | 9 |
| CC | GO:0000792 | heterochromatin | 8/604 | 78/19869 | 0.00251826 | 0.09889539 | 8 |
| CC | GO:0042641 | actomyosin | 8/604 | 78/19869 | 0.00251826 | 0.09889539 | 8 |
| CC | GO:0055038 | recycling endosome membrane | 9/604 | 97/19869 | 0.0027767 | 0.10125572 | 9 |
| CC | GO:0042589 | zymogen granule membrane | 3/604 | 11/19869 | 0.00384293 | 0.12608742 | 3 |
| CC | GO:0030532 | small nuclear ribonucleoprotein complex | 9/604 | 105/19869 | 0.00472021 | 0.12608742 | 9 |
| CC | GO:0001650 | fibrillar center | 11/604 | 145/19869 | 0.00482373 | 0.12608742 | 11 |
| CC | GO:0030140 | trans-Golgi network transport vesicle | 5/604 | 37/19869 | 0.00497829 | 0.12608742 | 5 |
| CC | GO:0001725 | stress fiber | 7/604 | 70/19869 | 0.00530506 | 0.12608742 | 7 |
| CC | GO:0097517 | contractile actin filament bundle | 7/604 | 70/19869 | 0.00530506 | 0.12608742 | 7 |
| CC | GO:0030136 | clathrin-coated vesicle | 14/604 | 211/19869 | 0.00531245 | 0.12608742 | 14 |
| CC | GO:0005798 | Golgi-associated vesicle | 8/604 | 89/19869 | 0.00568043 | 0.12608742 | 8 |
| CC | GO:0045171 | intercellular bridge | 8/604 | 89/19869 | 0.00568043 | 0.12608742 | 8 |
| CC | GO:0005681 | spliceosomal complex | 13/604 | 195/19869 | 0.00681535 | 0.14132465 | 13 |
| CC | GO:0005684 | U2-type spliceosomal complex | 8/604 | 92/19869 | 0.00692054 | 0.14132465 | 8 |
| CC | GO:0055037 | recycling endosome | 13/604 | 198/19869 | 0.00770915 | 0.14977931 | 13 |
| CC | GO:0042588 | zymogen granule | 3/604 | 14/19869 | 0.00792132 | 0.14977931 | 3 |
| CC | GO:0005912 | adherens junction | 12/604 | 179/19869 | 0.00875276 | 0.15754272 | 12 |
| CC | GO:0120114 | Sm-like protein family complex | 9/604 | 116/19869 | 0.00894908 | 0.15754272 | 9 |
| CC | GO:0016607 | nuclear speck | 22/604 | 419/19869 | 0.00955003 | 0.15942932 | 22 |
| CC | GO:0005847 | mRNA cleavage and polyadenylation specificity factor complex | 3/604 | 15/19869 | 0.00968081 | 0.15942932 | 3 |
| CC | GO:0032432 | actin filament bundle | 7/604 | 79/19869 | 0.01016826 | 0.16222385 | 7 |
| CC | GO:0005604 | basement membrane | 8/604 | 99/19869 | 0.01060014 | 0.16306929 | 8 |
| CC | GO:0033116 | endoplasmic reticulum-Golgi intermediate compartment membrane | 7/604 | 80/19869 | 0.01086008 | 0.16306929 | 7 |
| CC | GO:0046540 | U4/U6 x U5 tri-snRNP complex | 5/604 | 45/19869 | 0.01144696 | 0.16697065 | 5 |
| CC | GO:0097526 | spliceosomal tri-snRNP complex | 5/604 | 46/19869 | 0.01253019 | 0.17769426 | 5 |
| CC | GO:0071004 | U2-type prespliceosome | 3/604 | 17/19869 | 0.0138314 | 0.18582357 | 3 |
| CC | GO:0071010 | prespliceosome | 3/604 | 17/19869 | 0.0138314 | 0.18582357 | 3 |
| CC | GO:0005938 | cell cortex | 17/604 | 312/19869 | 0.01520993 | 0.19910437 | 17 |
| MF | GO:0045296 | cadherin binding | 28/602 | 333/18432 | 5.11E-06 | 0.00331255 | 28 |
| MF | GO:0030020 | extracellular matrix structural constituent conferring tensile strength | 8/602 | 41/18432 | 4.55E-05 | 0.0116577 | 8 |
| MF | GO:0005201 | extracellular matrix structural constituent | 17/602 | 173/18432 | 5.39E-05 | 0.0116577 | 17 |

The filtering criteria of p value < 0.05 and q value < 0.2.

**Supplementary Table 5. The results of Kyoto Encyclopedia of Genes and Genomes (KEGG) pathway analysis**

| **ID** | **Description** | **GeneRatio** | **BgRatio** | **pvalue** | **qvalue** | **Count** |
| --- | --- | --- | --- | --- | --- | --- |
| hsa05417 | Lipid and atherosclerosis | 16/236 | 215/8292 | 0.00040536 | 0.05215554 | 16 |
| hsa03040 | Spliceosome | 13/236 | 156/8292 | 0.00048781 | 0.05215554 | 13 |
| hsa04622 | RIG-I-like receptor signaling pathway | 8/236 | 71/8292 | 0.00086624 | 0.05215554 | 8 |
| hsa05219 | Bladder cancer | 6/236 | 41/8292 | 0.00097284 | 0.05215554 | 6 |
| hsa05165 | Human papillomavirus infection | 20/236 | 331/8292 | 0.00117412 | 0.05215554 | 20 |
| hsa04210 | Apoptosis | 11/236 | 136/8292 | 0.0016738 | 0.06195985 | 11 |
| hsa05146 | Amoebiasis | 9/236 | 102/8292 | 0.00241497 | 0.06807284 | 9 |
| hsa04974 | Protein digestion and absorption | 9/236 | 103/8292 | 0.00258216 | 0.06807284 | 9 |
| hsa04064 | NF-kappa B signaling pathway | 9/236 | 104/8292 | 0.0027584 | 0.06807284 | 9 |
| hsa04141 | Protein processing in endoplasmic reticulum | 12/236 | 171/8292 | 0.00346191 | 0.07689075 | 12 |
| hsa05163 | Human cytomegalovirus infection | 14/236 | 225/8292 | 0.00489828 | 0.0989031 | 14 |
| hsa04621 | NOD-like receptor signaling pathway | 12/236 | 186/8292 | 0.00675544 | 0.12503498 | 12 |
| hsa04933 | AGE-RAGE signaling pathway in diabetic complications | 8/236 | 100/8292 | 0.00749866 | 0.12811484 | 8 |
| hsa05142 | Chagas disease | 8/236 | 102/8292 | 0.00842064 | 0.12961578 | 8 |
| hsa03250 | Viral life cycle - HIV-1 | 6/236 | 63/8292 | 0.00875367 | 0.12961578 | 6 |
| hsa05130 | Pathogenic Escherichia coli infection | 12/236 | 197/8292 | 0.01046054 | 0.14520879 | 12 |
| hsa04211 | Longevity regulating pathway | 7/236 | 89/8292 | 0.01319566 | 0.16478913 | 7 |
| hsa05160 | Hepatitis C | 10/236 | 157/8292 | 0.01403686 | 0.16478913 | 10 |
| hsa04270 | Vascular smooth muscle contraction | 9/236 | 134/8292 | 0.01409689 | 0.16478913 | 9 |
| hsa05135 | Yersinia infection | 9/236 | 137/8292 | 0.01609704 | 0.17876183 | 9 |
| hsa05162 | Measles | 9/236 | 139/8292 | 0.01754153 | 0.18552696 | 9 |
| hsa04623 | Cytosolic DNA-sensing pathway | 6/236 | 75/8292 | 0.019606 | 0.19246802 | 6 |
| hsa05133 | Pertussis | 6/236 | 76/8292 | 0.02079749 | 0.19246802 | 6 |
| hsa05220 | Chronic myeloid leukemia | 6/236 | 76/8292 | 0.02079749 | 0.19246802 | 6 |
| hsa04071 | Sphingolipid signaling pathway | 8/236 | 121/8292 | 0.02188997 | 0.19263546 | 8 |
| hsa05134 | Legionellosis | 5/236 | 57/8292 | 0.02255022 | 0.19263546 | 5 |
| hsa05131 | Shigellosis | 13/236 | 247/8292 | 0.02398769 | 0.19732562 | 13 |
| hsa04330 | Notch signaling pathway | 5/236 | 59/8292 | 0.02576785 | 0.2043991 | 5 |
| hsa04144 | Endocytosis | 13/236 | 251/8292 | 0.02691197 | 0.20611343 | 13 |
| hsa04620 | Toll-like receptor signaling pathway | 7/236 | 104/8292 | 0.02853721 | 0.2088554 | 7 |
| hsa05169 | Epstein-Barr virus infection | 11/236 | 202/8292 | 0.02927648 | 0.2088554 | 11 |
| hsa04926 | Relaxin signaling pathway | 8/236 | 129/8292 | 0.03063367 | 0.2088554 | 8 |
| hsa05203 | Viral carcinogenesis | 11/236 | 204/8292 | 0.03115588 | 0.2088554 | 11 |
| hsa05205 | Proteoglycans in cancer | 11/236 | 205/8292 | 0.03212724 | 0.2088554 | 11 |
| hsa05171 | Coronavirus disease - COVID-19 | 12/236 | 232/8292 | 0.03306586 | 0.2088554 | 12 |
| hsa04150 | mTOR signaling pathway | 9/236 | 156/8292 | 0.03385239 | 0.2088554 | 9 |
| hsa04929 | GnRH secretion | 5/236 | 64/8292 | 0.03505107 | 0.21040612 | 5 |
| hsa04512 | ECM-receptor interaction | 6/236 | 88/8292 | 0.03905306 | 0.22495917 | 6 |
| hsa05145 | Toxoplasmosis | 7/236 | 112/8292 | 0.04032694 | 0.22495917 | 7 |
| hsa05221 | Acute myeloid leukemia | 5/236 | 67/8292 | 0.04149746 | 0.22495917 | 5 |
| hsa05161 | Hepatitis B | 9/236 | 162/8292 | 0.04152682 | 0.22495917 | 9 |
| hsa04668 | TNF signaling pathway | 7/236 | 114/8292 | 0.04370556 | 0.23112463 | 7 |
| hsa04920 | Adipocytokine signaling pathway | 5/236 | 69/8292 | 0.04616809 | 0.23545272 | 5 |
| hsa05222 | Small cell lung cancer | 6/236 | 92/8292 | 0.04686564 | 0.23545272 | 6 |
| hsa05202 | Transcriptional misregulation in cancer | 10/236 | 193/8292 | 0.04873571 | 0.23545272 | 10 |
| hsa04936 | Alcoholic liver disease | 8/236 | 142/8292 | 0.04949534 | 0.23545272 | 8 |

**Supplementary Table 6: The rank of hub genes via various of situations.**

| **Node-name** | **MCC** | **DMNC** | **MNC** | **Degree** | **EPC** | **BottleNeck** | **EcCentricity** | **Closeness** | **Radiality** | **Betweenness** | **Stress** |
| --- | --- | --- | --- | --- | --- | --- | --- | --- | --- | --- | --- |
| *hsa-miR-205-5p* | 15 | 0 | 1 | 15 | 12.725 | 43 | 0.07776 | 22.6619 | 3.40838 | 922.20476 | 1556 |
| *hsa-miR-149-3p* | 8 | 0 | 1 | 8 | 10.772 | 7 | 0.07776 | 18.12857 | 3.25286 | 474.69048 | 936 |
| *hsa-miR-449b-3p* | 7 | 0 | 1 | 7 | 4.286 | 8 | 0.10127 | 7 | 0.24593 | 42 | 42 |
| *hsa-miR-1224-5p* | 6 | 0 | 1 | 6 | 7.787 | 10 | 0.10886 | 15.71667 | 3.04551 | 646 | 1150 |
| *hsa-miR-3614-5p* | 6 | 0 | 1 | 6 | 11.831 | 42 | 0.10886 | 18.35 | 3.51206 | 795.12857 | 1722 |
| *hsa-miR-202-5p* | 5 | 0 | 1 | 5 | 3.385 | 6 | 0.07595 | 5 | 0.19747 | 20 | 20 |
| *hsa-miR-7974* | 5 | 0 | 1 | 5 | 3.385 | 6 | 0.07595 | 5 | 0.19747 | 20 | 20 |
| *hsa-miR-135b-3p* | 4 | 0 | 1 | 4 | 7.631 | 4 | 0.10886 | 14.65 | 3.04551 | 231.64286 | 460 |
| *hsa-miR-1298-5p* | 4 | 0 | 1 | 4 | 2.931 | 5 | 0.06329 | 4 | 0.17405 | 12 | 12 |
| *hsa-miR-934* | 4 | 0 | 1 | 4 | 4.482 | 4 | 0.07776 | 11.48095 | 2.16426 | 240 | 408 |
| *hsa-miR-885-5p* | 3 | 0 | 1 | 3 | 2.429 | 4 | 0.05063 | 3 | 0.1519 | 6 | 6 |
| *hsa-miR-4705* | 3 | 0 | 1 | 3 | 2.401 | 4 | 0.05063 | 3 | 0.1519 | 6 | 6 |
| *hsa-miR-31-3p* | 3 | 0 | 1 | 3 | 8.24 | 3 | 0.07776 | 13.6119 | 2.91591 | 92.33333 | 210 |
| *AKAP11* | 3 | 0 | 1 | 3 | 11.395 | 22 | 0.09072 | 18.7 | 3.52502 | 551.11429 | 1098 |
| *GMFB* | 3 | 0 | 1 | 3 | 10.499 | 21 | 0.09072 | 17.45 | 3.39542 | 347.35238 | 752 |
| *hsa-circ-0000994* | 3 | 0 | 1 | 3 | 9.437 | 37 | 0.13608 | 16.33333 | 3.34358 | 741.64286 | 1444 |
| *PAFAH1B2* | 2 | 0 | 1 | 2 | 8.336 | 3 | 0.09072 | 13.53333 | 3.03255 | 46.64762 | 132 |
| *KMO* | 2 | 0 | 1 | 2 | 7.766 | 1 | 0.09072 | 14.31667 | 3.08439 | 114 | 264 |
| *hsa-circ-0000439* | 2 | 0 | 1 | 2 | 5.263 | 5 | 0.09072 | 12.43333 | 2.61784 | 304 | 528 |
| *C5orf51* | 2 | 0 | 1 | 2 | 9.021 | 1 | 0.09072 | 14.78333 | 3.16215 | 79.24286 | 244 |
| *hsa-miR-670-3p* | 2 | 0 | 1 | 2 | 1.956 | 3 | 0.03797 | 2 | 0.13291 | 2 | 2 |
| *PGRMC1* | 1 | 0 | 1 | 1 | 2.914 | 1 | 0.06804 | 8.62024 | 1.63291 | 0 | 0 |
| *CMTM6* | 1 | 0 | 1 | 1 | 2.801 | 1 | 0.05063 | 4 | 0.15913 | 0 | 0 |
| *RBM26* | 1 | 0 | 1 | 1 | 2.22 | 1 | 0.03165 | 2.5 | 0.12658 | 0 | 0 |
| *CD164* | 1 | 0 | 1 | 1 | 2.437 | 1 | 0.03797 | 3 | 0.13671 | 0 | 0 |
| *CCNI* | 1 | 0 | 1 | 1 | 2.885 | 1 | 0.05063 | 4 | 0.15913 | 0 | 0 |
| *TNPO1* | 1 | 0 | 1 | 1 | 6.771 | 1 | 0.06804 | 13.83452 | 2.87703 | 0 | 0 |
| *EHF* | 1 | 0 | 1 | 1 | 2.228 | 1 | 0.03165 | 2.5 | 0.12658 | 0 | 0 |
| *U2SURP* | 1 | 0 | 1 | 1 | 2.785 | 1 | 0.05063 | 4 | 0.15913 | 0 | 0 |
| *NAGPA* | 1 | 0 | 1 | 1 | 2.42 | 1 | 0.03797 | 3 | 0.13671 | 0 | 0 |
| *IL6ST* | 1 | 0 | 1 | 1 | 7.209 | 1 | 0.06804 | 13.83452 | 2.87703 | 0 | 0 |
| *TMEM106B* | 1 | 0 | 1 | 1 | 6.584 | 1 | 0.09072 | 12.61667 | 2.98071 | 0 | 0 |
| *CLIP2* | 1 | 0 | 1 | 1 | 2.333 | 1 | 0.03797 | 3 | 0.13671 | 0 | 0 |
| *NHLRC2* | 1 | 0 | 1 | 1 | 6.852 | 1 | 0.06804 | 13.83452 | 2.87703 | 0 | 0 |
| *TRA2A* | 1 | 0 | 1 | 1 | 6.73 | 1 | 0.06804 | 13.83452 | 2.87703 | 0 | 0 |
| *MEX3D* | 1 | 0 | 1 | 1 | 1.937 | 1 | 0.02532 | 2 | 0.11814 | 0 | 0 |
| *hsa-circ-0000467* | 1 | 0 | 1 | 1 | 1.915 | 1 | 0.02532 | 2 | 0.11814 | 0 | 0 |
| *KIF1C* | 1 | 0 | 1 | 1 | 1.975 | 1 | 0.02532 | 2 | 0.11814 | 0 | 0 |
| *CPSF6* | 1 | 0 | 1 | 1 | 7.017 | 1 | 0.06804 | 13.83452 | 2.87703 | 0 | 0 |
| *GABPA* | 1 | 0 | 1 | 1 | 7.088 | 1 | 0.06804 | 13.83452 | 2.87703 | 0 | 0 |
| *hsa-circ-0003270* | 1 | 0 | 1 | 1 | 4.705 | 1 | 0.09072 | 11.01667 | 2.51417 | 0 | 0 |
| *hsa-circ-0002058* | 1 | 0 | 1 | 1 | 4.582 | 1 | 0.09072 | 11.01667 | 2.51417 | 0 | 0 |
| *hsa-circ-0001726* | 1 | 0 | 1 | 1 | 4.426 | 1 | 0.09072 | 11.01667 | 2.51417 | 0 | 0 |
| *PAPOLA* | 1 | 0 | 1 | 1 | 4.513 | 1 | 0.09072 | 11.01667 | 2.51417 | 0 | 0 |
| *GOLT1B* | 1 | 0 | 1 | 1 | 2.787 | 1 | 0.06804 | 8.62024 | 1.63291 | 0 | 0 |
| *SHOC2* | 1 | 0 | 1 | 1 | 2.927 | 1 | 0.05063 | 4 | 0.15913 | 0 | 0 |
| *MLLT1* | 1 | 0 | 1 | 1 | 2.476 | 1 | 0.03797 | 3 | 0.13671 | 0 | 0 |
| *TMED10* | 1 | 0 | 1 | 1 | 6.148 | 1 | 0.06804 | 12.16786 | 2.72152 | 0 | 0 |
| *LYSMD3* | 1 | 0 | 1 | 1 | 6.726 | 1 | 0.06804 | 13.83452 | 2.87703 | 0 | 0 |
| *PTP4A1* | 1 | 0 | 1 | 1 | 6.919 | 1 | 0.06804 | 13.83452 | 2.87703 | 0 | 0 |
| *CLTA* | 1 | 0 | 1 | 1 | 1.901 | 1 | 0.02532 | 2 | 0.11814 | 0 | 0 |
| *SESN3* | 1 | 0 | 1 | 1 | 2.454 | 1 | 0.03797 | 3 | 0.13671 | 0 | 0 |
| *LIMS1* | 1 | 0 | 1 | 1 | 4.691 | 1 | 0.09072 | 10.68333 | 2.51417 | 0 | 0 |
| *SRSF11* | 1 | 0 | 1 | 1 | 2.737 | 1 | 0.05063 | 4 | 0.15913 | 0 | 0 |
| *hsa-circ-0005616* | 1 | 0 | 1 | 1 | 1.918 | 1 | 0.02532 | 2 | 0.11814 | 0 | 0 |
| *KIAA2013* | 1 | 0 | 1 | 1 | 1.906 | 1 | 0.02532 | 2 | 0.11814 | 0 | 0 |
| *RPL15* | 1 | 0 | 1 | 1 | 5.999 | 1 | 0.06804 | 12.16786 | 2.72152 | 0 | 0 |
| *ETNK1* | 1 | 0 | 1 | 1 | 6.848 | 1 | 0.06804 | 13.83452 | 2.87703 | 0 | 0 |
| *hsa-circ-0001535* | 1 | 0 | 1 | 1 | 4.722 | 1 | 0.06804 | 10.20119 | 2.38457 | 0 | 0 |
| *hsa-circ-0007334* | 1 | 0 | 1 | 1 | 2.396 | 1 | 0.03797 | 3 | 0.13671 | 0 | 0 |
| *PRR12* | 1 | 0 | 1 | 1 | 2.366 | 1 | 0.03797 | 3 | 0.13671 | 0 | 0 |
| *EDNRA* | 1 | 0 | 1 | 1 | 5.87 | 1 | 0.06804 | 12.16786 | 2.72152 | 0 | 0 |
| *ZNF680* | 1 | 0 | 1 | 1 | 4.188 | 1 | 0.09072 | 10.68333 | 2.51417 | 0 | 0 |
| *RAB14* | 1 | 0 | 1 | 1 | 6.898 | 1 | 0.06804 | 13.83452 | 2.87703 | 0 | 0 |
| *CASD1* | 1 | 0 | 1 | 1 | 6.654 | 1 | 0.06804 | 13.83452 | 2.87703 | 0 | 0 |
| *hsa-circ-0004587* | 1 | 0 | 1 | 1 | 2.112 | 1 | 0.03165 | 2.5 | 0.12658 | 0 | 0 |
| *VPS13C* | 1 | 0 | 1 | 1 | 2.203 | 1 | 0.03165 | 2.5 | 0.12658 | 0 | 0 |
| *TTC14* | 1 | 0 | 1 | 1 | 2.895 | 1 | 0.06804 | 8.62024 | 1.63291 | 0 | 0 |
| *SRSF10* | 1 | 0 | 1 | 1 | 6.934 | 1 | 0.06804 | 13.83452 | 2.87703 | 0 | 0 |
| *hsa-circ-0007509* | 1 | 0 | 1 | 1 | 7.076 | 1 | 0.06804 | 13.83452 | 2.87703 | 0 | 0 |
| *hsa-circ-0099549* | 1 | 0 | 1 | 1 | 2.698 | 1 | 0.05063 | 4 | 0.15913 | 0 | 0 |
| *ZDHHC21* | 1 | 0 | 1 | 1 | 2.671 | 1 | 0.05063 | 4 | 0.15913 | 0 | 0 |
| *hsa-circ-0001727* | 1 | 0 | 1 | 1 | 5.778 | 1 | 0.06804 | 12.16786 | 2.72152 | 0 | 0 |
| *CPM* | 1 | 0 | 1 | 1 | 5.709 | 1 | 0.06804 | 12.16786 | 2.72152 | 0 | 0 |
| *hsa-circ-0000069* | 1 | 0 | 1 | 1 | 1.705 | 1 | 0.01899 | 1.5 | 0.11392 | 0 | 0 |
| *GNA11* | 1 | 0 | 1 | 1 | 1.713 | 1 | 0.01899 | 1.5 | 0.11392 | 0 | 0 |
| *hsa-circ-0032649* | 1 | 0 | 1 | 1 | 2.341 | 1 | 0.03797 | 3 | 0.13671 | 0 | 0 |
| *hsa-circ-0072732* | 1 | 0 | 1 | 1 | 2.387 | 1 | 0.03797 | 3 | 0.13671 | 0 | 0 |
| *TMED2* | 1 | 0 | 1 | 1 | 2.386 | 1 | 0.03797 | 3 | 0.13671 | 0 | 0 |
